# A Review of Diamond Materials and Applications in Power Semiconductor Devices

**DOI:** 10.3390/ma17143437

**Published:** 2024-07-11

**Authors:** Feiyang Zhao, Yongjie He, Bin Huang, Tianyi Zhang, Hao Zhu

**Affiliations:** 1 School of Microelectronics, Fudan University, Shanghai 200433, China; 23212020203@m.fudan.edu.cn (F.Z.); 22212020007@m.fudan.edu.cn (Y.H.); 23212020081@m.fudan.edu.cn (B.H.); 23212020196@m.fudan.edu.cn (T.Z.); 2 National Integrated Circuit Innovation Center, Shanghai 201203, China

**Keywords:** diamond, Schottky barrier diodes (SBDs), field-effect transistors (FETs), power devices

## Abstract

Diamond is known as the ultimate semiconductor material for electric devices with excellent properties such as an ultra-wide bandgap (5.47 eV), high carrier mobility (electron mobility 4000 cm^2^/V·s, hole mobility 3800 cm^2^/V·s), high critical breakdown electric field (20 MV/cm), and high thermal conductivity (22 W/cm·K), showing good prospects in high-power applications. The lack of n-type diamonds limits the development of bipolar devices; most of the research focuses on p-type Schottky barrier diodes (SBDs) and unipolar field-effect transistors (FETs) based on terminal technology. In recent years, breakthroughs have been made through the introduction of new structures, dielectric materials, heterogeneous epitaxy, etc. Currently, diamond devices have shown promising applications in high-power applications, with a BV of 10 kV, a BFOM of 874.6 MW/cm^2^, and a current density of 60 kA/cm^2^ already realized. This review summarizes the research progress of diamond materials, devices, and specific applications, with a particular focus on the development of SBDs and FETs and their use in high-power applications, aiming to provide researchers with the relevant intuitive parametric comparisons. Finally, the paper provides an outlook on the parameters and development directions of diamond power devices.

## 1. Introduction

Power semiconductor devices are core components in electronic power systems used in almost all electronic manufacturing industries. Typical application areas include consumer electronics, mobile communications, electronic equipment, etc. [1]. The type of semiconductor has played a critical role in specific applications like power devices [2]. For example, Si is still the most widely used material in semiconductors and integrated circuits in the field, and more than ninety percent of the devices use silicon as a material. However, as the device size shrinks, the performance of Si gradually fails to meet the requirements of a variety of applications. This is due to its narrow bandgap (1.12 eV), lower breakdown field (~3 × 10^5^ MV/cm) and saturation speed (~10^7^ cm/s), and other defects, for example, the specific on-resistance of silicon-based power devices is proportional to the 2.5 power of the breakdown voltage, which constrains the improvement of the power figure of merit. All these defects limit its application in high-voltage, high-power occasions [3,4,5]. Some high-carrier-mobility semiconductors like indium phosphide (InP) and gallium arsenide (GaAs) have alternatively been used in high-performance microwave and millimeter-wave devices and light-emitting electronic devices [6]. Nevertheless, GaAs and InP still have problems such as high cost, material scarcity, and high toxicity, and their narrow bandgap (1.4 eV) makes them unsuitable in advanced power device applications.

As the need for ever more efficient power electronics grows, semiconductors with wide bandgaps are becoming increasingly important [7,8]. As representatives, silicon carbide (SiC) and gallium nitride (GaN) have been widely investigated and implemented in various power device applications [9,10,11]. The bandgap of SiC is about three times that of Si, making it suitable for fabricating high-frequency, high-voltage, and high-power devices. Specifically, the 4H-SiC with a relatively larger bandgap, lower anisotropies, and higher mobility has been adopted for commercial product manufacturing [12,13]. However, it still has drawbacks like relatively lower mobility than most semiconductor materials, and the poor interface quality of 4H-SiC/SiO_2_ with its high interface trap density has urged significant process improvements. This is because the natural oxide of SiC is also SiO_2_, and the C-O-Si bonding at the interface after oxidation induces more interfacial states [14]. GaN is another wide bandgap semiconductor material with high electron mobility and high electron saturation rate, which has a wide range of applications in the field of optoelectronics including light-emitting diodes (LEDs) [15] and laser diodes [16]. However, GaN devices also suffer from fluctuations in performance parameters like on-resistance and output capacitance in high-frequency operations [17]. GaN HEMTs also exhibit degraded robustness when subjected to high voltages, which limits their applications in high-frequency and high-temperature fields [18].

Recent years have witnessed the rapid development of the next-generation ultra-wide bandgap (4.9~6.4 eV) semiconductor materials, mainly including gallium oxide (Ga_2_O_3_) [19,20], diamond [21,22], and aluminum nitride (AlN) [23]. Among them, AlN has excellent dielectric properties and chemical stability. In particular, its coefficient of thermal expansion is similar to that of silicon and matches the characteristics of the semiconductor package making it a very promising substrate material. However, since the highest thermal conductivity can only be 260 W/(m·K), as semiconductor packages require more and more heat dissipation, the AlN material has a certain development bottleneck. Therefore, at present, the main research has been carried out on Ga_2_O_3_ and diamond.

Ga_2_O_3_ has a bandgap of 4.9 eV with its ultraviolet absorption edge situated between 260 nm and 280 nm, which makes Ga_2_O_3_ a rare and ideal material for solar blind UV detection [24]. However, the drawback of this material is that the thermal conductivity is very low, about 1/8 of GaN and 1/5 of Si, which limits the device’s performance and reduces the device’s lifetime. In addition, there is a lack of p-type β-Ga2O3 in nature and it is not easy to achieve p-type doping. It is presumed to be related to the presence of oxygen vacancies, which capture free electrons, making it difficult to form natural p-type Ga_2_O_3_ [25].

Therefore, another material, diamond, which has a larger bandgap, higher thermal conductivity, and makes up for the lack of p-type devices, has gained significant interest. This review introduces the exceptional physical properties of diamond semiconductors and gives a comprehensive review of the research progress and applications of diamond-based power devices up to the year 2024. Section 2 summarizes the properties and potential of diamond materials, as well as the technical approaches utilized in diamond-based semiconductor devices, such as diamond growth, doping means, etching, and terminal processing. Section 3 and Section 4 review the applications of diamond in various electronic devices and device characterizations, such as diodes, field-effect transistors, and bipolar transistors. Section 5 discusses the circuit-level application of diamond-based devices, followed by a summary and prospects in Section 6.

## 2. Diamond Materials and Processes for Power Applications

### 2.1. Diamond Properties

A certain amount of natural diamond exists in nature and is of great value. Synthetic diamonds are used in industry. The application of diamond in semiconductor materials can be traced back to the 1950s. In 1958, M. D. Bell et al. [26] reported the use of natural p-type diamond in the fabrication of power diodes, due to the challenging synthesis and effective doping of diamond. Until a breakthrough in the 21st century (which will be discussed in the following chapters), the research progress of both materials and devices is relatively slow, limiting its adoption compared to the rapid advancements in silicon technology.

**Figure 1:**
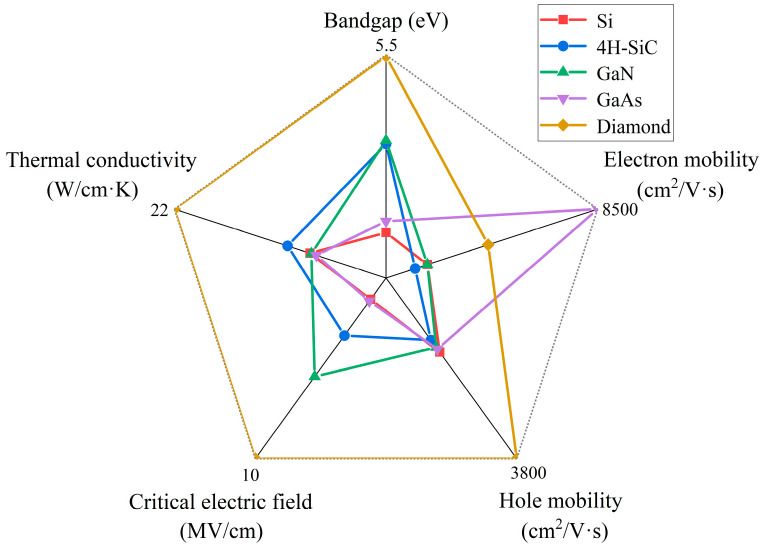
Comparison of diamond properties with other materials. Diamond has the largest bandgap, breakdown electric field, thermal conductivity, and hole mobility. Besides GaAs, diamond has the highest electron mobility.

A comparison of the physical parameters of diamond with other semiconductor materials is given in Figure 1. The advantages and disadvantages of Si, 4H-SiC, and GaN have already been mentioned in Section 1. For GaAs, which is a high electron mobility material suitable for microwave devices and high-speed digital circuits, however, its bandgap and critical electric field are only slightly higher than those of Si, at 1.42 eV and 0.4 MV/cm, respectively. And its thermal conductivity is at 0.45 W/cm·k. These defects limit their use in high-temperature and high-pressure areas [27].

Notably, diamond exhibits superior properties in almost every aspect. Due to the large band gap (5.47 eV), the high theoretical maximum breakdown field strength (20 MV/cm), and high electron and hole mobility, diamond is a promising candidate for applications in high-performance and high-power electronics. In addition, compared with other semiconducting materials, diamond material has a saturation drift rate of 1.5 × 10^7^ cm/s for electrons and 1.05 × 10^7^ cm/s for holes without breakdown [28]. The thermal conductivity of a single-crystal diamond can reach 22 W/cm·K, and the thermal conductivity of a polycrystalline diamond can also achieve 20 W/cm·K [29,30], making it attractive in applications with specific requirements of thermal dissipation.

**Figure 2:**
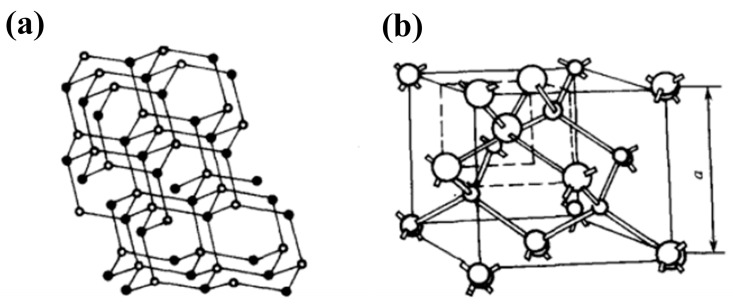
Schematic representation of the diamond (**a**) crystal structure and (**b**) crystal cell. Each carbon atom forms a covalent bond with 4 other carbon atoms to form a positive tetrahedron. The strong C-C bonds make diamond hard and have a high melting point. All valence electrons are confined by the covalent bonds, which is why diamond does not conduct electricity [31].

For the diamond material, each carbon atom within the diamond cell is connected to the other four atoms by covalent bonds. The C-C bonds are bonded heterogeneously by sp^3^ to form an orthotetrahedron, which belongs to face-centered cubic crystals, and its structure is shown in Figure 2 [31]. The lattice constant is 3.667 Å, and the C-C bond length is 1.54 Å with a bond angle of 109°28′. Due to the compact and symmetrical arrangement of carbon atoms in a diamond, the covalent bonds are exceptionally strong. This strong bonding, combined with the absence of free electrons, enables remarkable physical and chemical stability for diamonds. Therefore, diamond is a very good insulator, with a bandgap width of up to 5.47 eV. Diamond has a small atomic weight, low defect density, and high average free path of phonons. Phonons can propagate over long distances without colliding with other phonons and lattice defects, thus possessing high thermal conductivity. Similarly, carrier scattering with phonons and defects are reduced, which makes diamond also have very high carrier mobility.

Diamond has three primary crystallographic orientations: (100), (110), and (111). These orientations significantly impact material and process properties like surface roughness, dopant incorporation, surface structure, cutting difficulty, and material price [32]. Among them, (100) and (001) are the equivalent crystallographic orientations of diamonds, both of which have the same structural properties. Nowadays, diamond epitaxial growth primarily utilizes the (100) crystallographic orientation due to its ease of growth and reduced processing difficulty [33]. The advantages of (110) and (111) homoepitaxial diamonds are that they offer a high degree of structural sym metry and can achieve high doping with a high density of atoms in the vertical direction.

**Figure 3:**
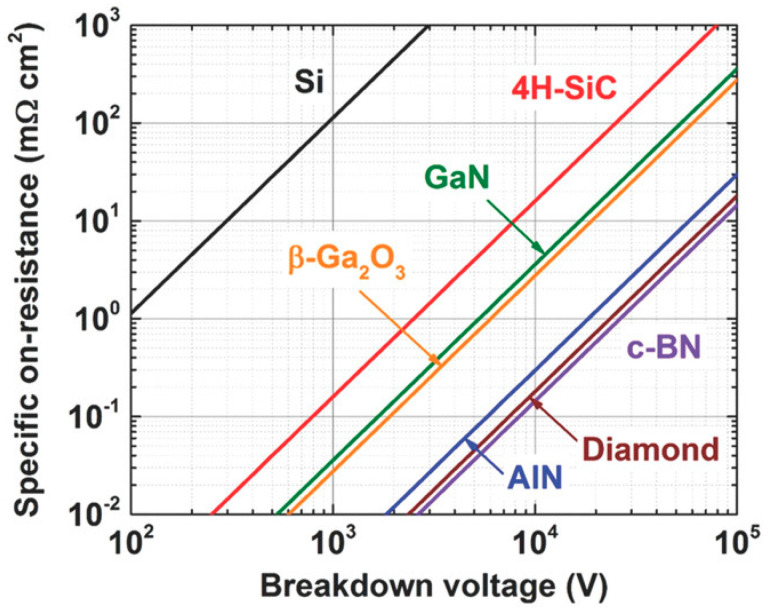
BFOM contours for conventional, wide-band, and ultra-wide-band semiconductors [34]. The BFOM of diamond far exceeds that of currently dominant semiconductor materials such as Si and 4H-SiC, and also has advantages over Ga_2_O_3_, which is also a UWBG material.

Diamond materials have expanded the application areas of power devices. The Baliga figure of merit (BFOM), which is widely used to judge the performance of power devices, is defined as follows [12]:

(1) $$BFOM=\frac{{BV}^{2}}{R_{on}}$$

where BV is the breakdown voltage and R_on_ is the specific on-resistance. The BFOM can demonstrate the device’s off-state performance and conduction power loss at low-frequency operation. The more significant the value, the higher the reverse voltage the device can withstand in its off-state, and the higher the conductivity per unit area during turn-on [34]. Since both breakdown voltage and conductivity are related to the doping concentration depletion region width, and both depend on the critical breakdown electric field E_c_, the BFOM values of various materials can be characterized as equations related to E_c_. The magnitude of E_c_ for each material is dependent on the bandgap, which reveals that the ideal BFOM values of various materials are constant. For a given material, the breakdown voltage and specific on-resistance under ideal conditions are in one-to-one correspondence, and the contours of the BFOM are shown in Figure 3, with the values closer to the lower right region representing higher BFOM [34].

The high BFOM underscores the exceptional potential of diamond power semiconductors for high-voltage and high-power applications. This potential has aroused recognition in many countries that diamond power devices can be critical components for next-generation electronics in both civilian and military applications. The U.S. Department of Defense Advanced Research Projects Agency launched the “near-junction thermal transport” project in 2011, which was dedicated to using high thermal conductivity diamond substrate development with the higher output power and smaller area of GaN microwave chips [35]. The United States AKHAN [36] has developed a diameter of 300 mm polycrystalline diamond substrate in the diamond substrate. Japan’s Adamant Namiki Precision Gemstone Corporation and the Saga University research team also announced the mass production of a 55 mm diameter single-crystal diamond substrate. At the same time, the University of Augsburg, Germany, reported a diameter of 92 mm heterogeneous epitaxial single-crystal diamond substrate [37]. The microwave power device developed by Waseda University in Japan has achieved a microwave output power density of 3.8 W/mm at 1 GHz [38]. The power device reported by Saga University in Japan has achieved a Baliga superiority of 875 MW/cm^2^ and a BV of more than 2500 V [39]. In addition to the synthesis technology, doping technology, and mass materials production, great interests have been triggered focusing on research and exploration in high-end manufacturing and cutting-edge high-tech fields. For example, Prof. Zhang’s team at Shanghai Jiaotong University has established a chemical gas phase surface reaction condition and growth model on the diamond generation process to theoretically explain the diamond’s surface growth morphology rate under chemical gas phase conditions [40,41]. Xi’an University of Electronic Science and Technology (XUEST) reported hydrogen-terminated diamond devices using a barium fluoride (BaF_2_) dielectric material, which achieved high electron mobility [42].

### 2.2. Diamond Substrate and Growth

Driven by the limitations of natural diamond reserves for the demanding requirements of the semiconductor industry, synthetic diamond production has become the primary source. There are two main methods for synthesizing diamonds: the high-temperature and high-pressure method (HPHT) [43] and microwave plasma chemical vapor deposition (MPCVD) [44].

The HPHT method replicates the extreme pressure and temperature conditions (typically 5.5–8.0 GPA and 1000–1400 °C) found deep within the Earth’s mantle, essentially recreating the natural environment where diamonds form. The key to the preparation of diamonds by the HPHT method is to simulate the growth environment of natural diamonds, and the focus and difficulty of the current research are how to simulate the natural diamond growth environment as much as possible. Today’s research and development are directed towards exploring different systems of growth environments, with the main environmental systems being C-N, C-O, C-H, C-H_2_O, C–silicate, and so on. Researchers have found that the transformation of graphite to diamond is favored in water-enriched environments [45,46]. However, the size of single-crystal diamonds obtained by the HPHT method is generally smaller than 10 mm, which limits the application of diamonds in many areas [47]. The more dominant method today is microwave plasma chemical vapor deposition (MPCVD).

**Figure 4:**
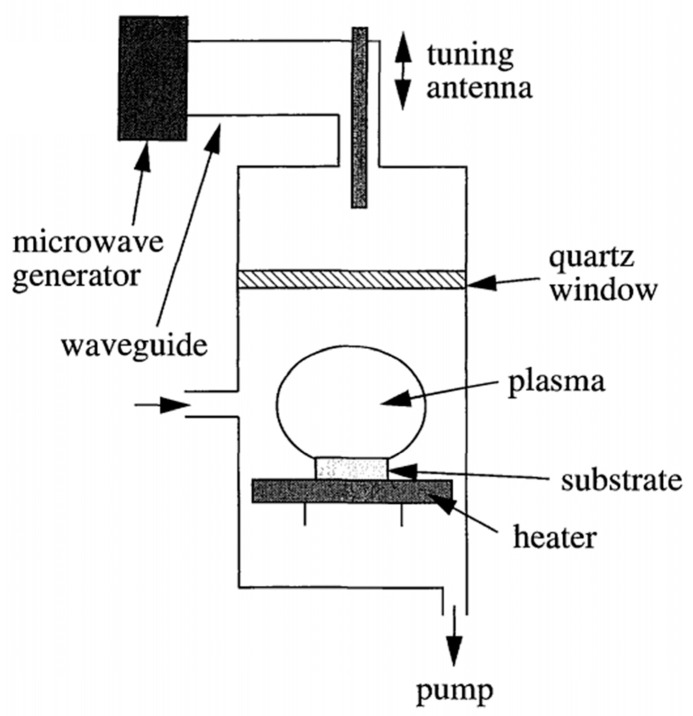
Schematic diagram of MPCVD equipment structure. The microwave generator generates energy, the tunneling antenna is used to transmit the energy from the waveguide to the inside of the reactor, and the quartz window is used to insulate the air. Below is the reaction chamber for the plasma treatment and heating of the substrate [49].

MPCVD is developed based on the CVD (chemical vapor deposition) technique, which is a versatile method for creating thin films by introducing gaseous precursors that undergo chemical reactions and physical changes on a substrate’s surface. MPCVD leverages this concept but utilizes microwave plasma to control the deposition process. This allows for precise control over factors like temperature, pressure, and the choice of precursor gas, ultimately leading to the formation of a diamond film on the substrate. In 1968, the Soviet scientists Derjaguin et al. [48] first used CVD to deposit diamond films on non-diamond substrates, but the speed was slow (only 250 μm/h). In 1983, the Japanese scientists M. Kamo et al. [44] successfully achieved the epitaxial growth of diamond films for the first time using the MPCVD method. Since then, MPCVD has become the mainstream method for preparing diamond films, and its equipment structure is shown in Figure 4 [49]. A mixture of methane and hydrogen is passed into the chamber. The plasma generated by the microwave power provides the activation energy for the decomposition of methane, and the carbon atoms obtained from the decomposition are deposited on the substrate surface. The etching rate of hydrogen on graphite is much higher than that of diamond, and the graphite is selectively etched away during the reaction process, ultimately leaving behind the diamond.

The factors that influence the MPCVD growth of diamonds are growth mode, air pressure, substrate temperature, etc. [50]. Different deposition air pressures will significantly change the plasma morphology and the content of active groups in the chamber, thus affecting the deposition rate area [51]. The substrate temperature has an impact on the morphology, quality, and growth rate of the deposited film, and the typical substrate temperature is generally between 800 and 1200 °C. It has been found that it is not easy to form high-quality diamond below 750 °C, and the growth rate is quite low with lots of defects. On the other hand, if the temperature is higher than 1200 °C, there is a tendency of graphitizing the diamond [52].

At present, the urgent problem of MPCVD growth diamond is how to further expand the size and improve the growth rate. Nad et al. [53] expanded the lateral growth area by using a closed substrate bracket, and the area was expanded to 1.7–2 times the seed crystal at the end of growth. Researchers also expanded the wafer size by assembling individual diamonds; Yamada et al. [54] at AIST in Japan successfully assembled 24 individual 10 mm × 10 mm single-crystal diamonds, grown via MPCVD, into a 2-inch wafer. In terms of growth rate, some studies have achieved high growth rates of 165 μm/h, 135 μm/h, and 70 μm/h by introducing N_2_, N_2_O, and CO_2_ into the growth process, respectively [55,56,57]. However, high growth rates in the gas-assisted case tend to bring more defects, which is a challenge for the current development.

**Figure 5:**
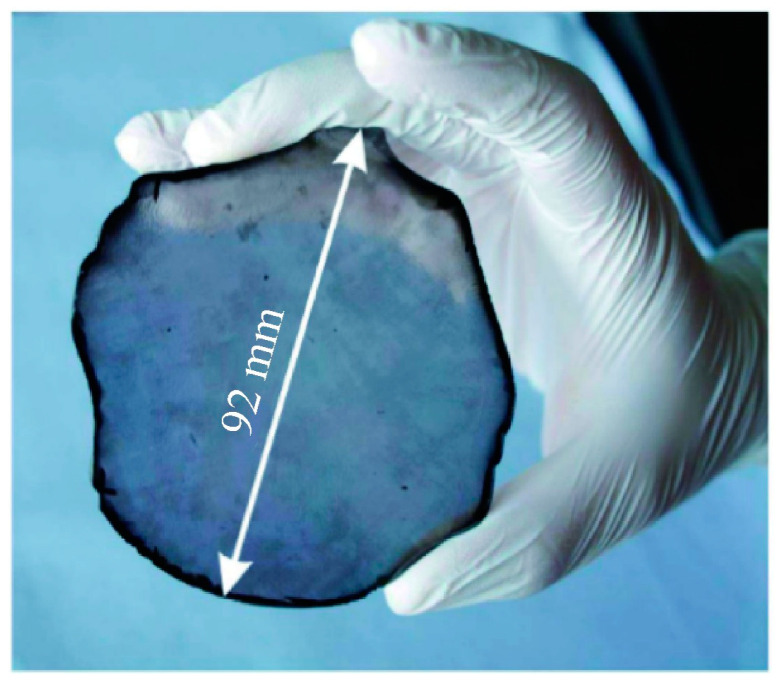
A 3.6-inch heterogeneous epitaxial single-crystal diamond substrate [59].

Beyond the previously mentioned homogeneous epitaxial growth, another approach tackles the challenge of undersized diamond crystals. Heterogeneous epitaxy offers a solution for achieving large-scale wafer growth, potentially reducing overall production costs [58]. Currently, the German Schreck team uses sapphire as a substrate and iridium (Ir) as a transition film to create a single-crystal diamond substrate, as shown in Figure 5 [59].

### 2.3. Diamond Etching

The main principle of diamond etching is to use gases with high chemical activity to react with diamond to produce volatile products, such as CO or CO_2_ [60]. In addition, the iron group metals [61], Fe, Co, and Ni, and their oxides can activate carbon atoms or provide them with reactive oxygen, thus promoting the oxidation of carbon. It is mentioned in the paper that transition metals have high catalytic activity and can promote the chemical reaction of C atoms. In addition, Raman spectroscopy shows that diamond can be transformed from a stable crystalline structure to a graphite structure that is easier to etch, and in some cases, transition metals can also form co-crystals with diamond, which can transform the metal into a liquid state by lowering the melting point of the metal and increasing the contact area.

There are many diamond etching methods, such as using reactive ion etching (RIE) [62,63], laser beam irradiation [64], inductively coupled plasma (ICP) [65,66], and so on. The diamond etching masks are generally SiO_2_ [67], Al [68,69], and Si_3_N_4_ [70,71].

**Figure 6:**
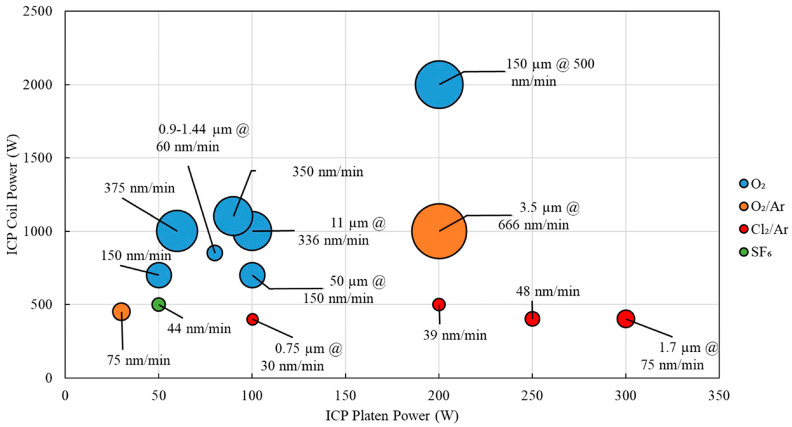
Etching rate versus ICP Coil/Platen power of the specified ICP-RIE process [60]. This figure gives the results of the etching rate of various gases as etching sources.

The critical parameters of the diamond etching process include the surface roughness after etching and the etching rate. A. Holland et al. [72] showed that the etching rate of pure O_2_ is medium, and the introduction of Ar has better homogeneity and reproducibility than O_2_. Y. Ando et al. [73] found that the roughness of the diamond surface after etching decreases with the increase in the CF_4_/O_2_ ratio, and corrosion resistance is the best when Al_2_O_3_ is used as the mask. The reason that gases can improve diamond etching is that reactive gases like CF_4_ can participate in the reaction to generate inactive compounds that act as a protective layer to reduce the occurrence of physical sputtering, thus reducing the consumption of oxygen atoms and allowing for an increase in the etching rate. The introduction of inert gases like Ar improves the gas pressure, which in turn improves the etching roughness and anisotropy and facilitates the realization of a smoother etching surface. The etch rate of gases is affected by a variety of factors, and Figure 6 visualizes the relationship between the most important gas sources and ICP power and etch rate [60].

Nowadays, diamond etching is mainly used for pseudo-vertical diode structures [74,75]. The reason for using etching is that many diamond substrates are of an undoped type and cannot directly produce back electrodes. Therefore, it is necessary to expose the epitaxial layers with different doping concentrations on the surface through etching. In addition, diamond is also used in trench diodes [76] and trench field-effect transistor structures [77], which helps to alleviate the electric field distribution. These examples will be mentioned later in the text.

### 2.4. Diamond Doping

Intrinsic diamond is an insulator with a bandgap of about 5.47 eV with no free electrons in the crystal [78]. How to achieve effective doping of diamond and thus control the material’s conductivity is a difficult task. Currently, the elements that can be used for doping are B, N, P, S, etc.

**Figure 7:**
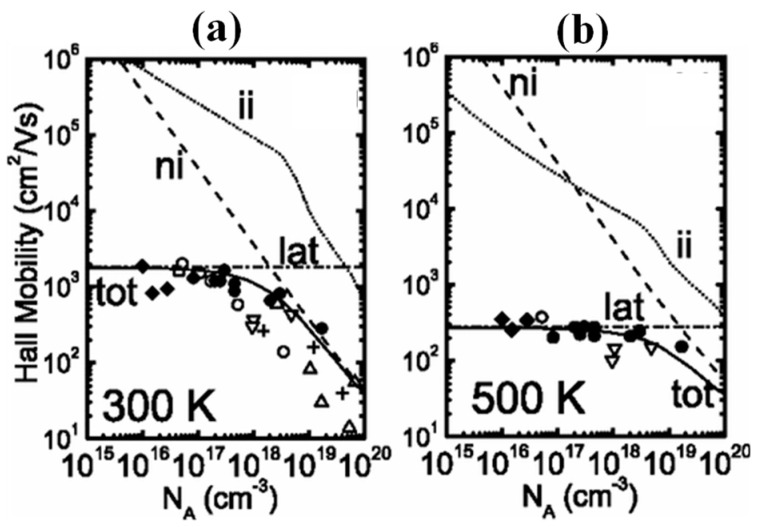
Hall mobility versus doping concentration at (**a**) 300 K and (**b**) 500 K [82].

The B element can be introduced in the diamond valence band at 0.37 eV above the principal energy level, commonly used in diamond p-type doping. A high crystal quality natural diamond containing a small amount of B impurities has a high activation energy, but the mobility at room temperature can reach 2200 cm^2^/V·s. As the concentration of B doping increases and the temperature increases, the activation energy decreases slightly, making it easier for valence band electrons to be excited into the conduction band, lowering the electrical resistivity. Still, the hole mobility in this process will be significantly degraded [79,80]. The relationship between the activation energy (EA) and doping concentration of a B-doped diamond is given by A. Deneuville et al. [81]. They found that when the B concentration exceeds 3 × 10^20^ cm^−3^, the activation energy becomes 0, and the superconducting property of the diamond is realized. Figure 7a,b give the relationship between Hall mobility and doping concentration at 300 K and 500 K, respectively [82]. When the doping concentration is higher than 10^19^, the mobility is as low as 100 cm^2^/V·s, and this defect limits the application of B-doped diamond in microwave devices.

The approach to achieve B-doping is by using MPCVD, where a boron source is introduced for doping during the growth of the film. This allows for high-quality doping of single-crystal diamond by suppressing the concentration of the residual gas in the MPCVD growth reaction chamber, which in turn reduces the probability of mixing impurities in the diamond. Different properties of diamond films can be obtained by regulating the gas source components. Barjon et al. [83] investigated the diamond films obtained by growth with different ratios (0.5–50 ppm) of B/C gas source, and the highest doping concentration achieved by increasing B/C in the (111) crystal direction is 9 × 10^21^ cm^−3^ [84]. In addition to the gas ratio, the plasma power density and cavity pressure modulation affect the growth rate and doping efficiency.

Diamonds can also be doped by annealing after ion implantation to repair the damage and activate the impurities, and the highest doping concentration can reach the order of 10^21^ cm^−3^ [85]. However, the annealing temperature required for diamond ion implantation is as high as 1700 °C [86]. This method is more challenging to achieve than MPCVD, which only requires a temperature of less than 1000 °C. From this perspective, MPCVD is more favorable to realize the p-type doping of diamond.

On the other hand, unfortunately, the n-type doping of diamond is difficult to achieve. Ion implantation introduces considerable lattice damage. Additionally, substituting nitrogen atoms for carbon atoms creates localized distortions in the lattice and vacancies nearby. These factors combine to elevate the activation energy, ultimately hindering conductivity [87]. P atom-doped diamonds also encounter the problems of high activation energy (~0.57 eV), low doping concentration (~10^19^ cm^−3^), and low activation efficiency [88,89].

It is challenging to achieve n-type doping of diamond with a single element, so researchers have proposed co-doping of multiple elements (B-O, B-S) to improve the doping process. It has been found that the introduction of B promotes the doping efficiency of O and S in diamond, although the mechanism has not yet been fully explored. Alkali metals such as lithium and sodium have been used in diamond doping as well. For example, interstitial Li atoms are used to displace C or fill in the gaps, thus exhibiting deep energy donor properties [90,91]. Su et al. [92] introduced a sulfur-mixed diamond film with electron mobility reaching 597 cm^2^/V·s at room temperature and a carrier concentration of 1.4 × 10^13^ cm^−3^. However, the film quality obtained by this method is low, and the principal components are unclear, so more studies are needed to explore the mechanisms.

### 2.5. Terminal Technology

Terminal technology, a technique for improving surface physical properties by modifying the diamond surface, has been proposed to enhance channel charge control. This is generally performed by plasma treatment with gases such as hydrogen, oxygen, and fluorine.

A highly conductive layer with negative electron affinity energy (NEA) exists on the surface of the H-treated diamond, which is conductive by holes [93]. This is the basis for developing H-terminated diamond field-effect transistors. There are two main explanations for the conductive mechanism of H-terminated diamonds. One is the transfer doping model, where the diamond on the surface of the hydrogen terminals adsorbs some molecules. These molecules exhibit differences in chemical potentials that can cause the electrons to spontaneously transfer to the surface layer, forming a two-dimensional hole gas (2DHG) [94]. The other model is based on spontaneous polarization. Owing to the electronegativity of the C atom (2.5) being higher than the H atom (2.1), the electron cloud between the C-H dipoles is closer to the C atom. Consequently, an electric field is formed inside the C-H dipole, directed from the H atom towards the C atom. Externally, the C atom acts as the negative charge center, and the H atom as the positive one. This results in the attraction of negatively charged adsorbates by the H atoms on the diamond surface, while the holes inside the diamond accumulate on the C atom side, ensuring an electrically neutral state. The thin layer of high-concentration holes on one side of the diamond is known as 2DHG [95].

The means of realizing the hydrogenation treatment on the diamond surface generally uses MPCVD equipment, which allows the diamond to receive hydrogen etching in the reaction chamber to form hydrogen terminals [96,97].

Oxygen termination is usually achieved using ultraviolet ozone (UV/O_3_) treatment [56], hot acid immersion, and plasma treatment [98] (such as RIE, etc.). In contrast to the NEA of the hydrogen terminal, the oxygen terminal has a positive electron affinity energy (PEA), which makes the diamond surface state more stable and unfavorable for use as a conductive channel in devices. They are commonly used for isolation between devices.

Like the principle of hydrogen and oxygen terminals, elements such as N, F, and Si can also modify the diamond surface depending on the electron affinity energy. The standard terminations and their electron affinity energies are given in Table 1 [93]. F and N have a PEA and are therefore similar to O terminals for performing device isolation, and Si terminals have an NEA, which can also induce a 2DHG. N and F terminals are carried out by treating the diamond surface using a plasma containing the gas of the corresponding element. Silicon terminals are implemented in various ways. For example, molecular beam deposition (MBD) can allow Si atoms to be deposited on the diamond surface and annealed under vacuum conditions [99,100]. Si thin films can be deposited by magnetron sputtering and then annealed under vacuum to form Si terminals [101]. The principle of terminal-induced formation of 2DHG and related studies will be discussed in more detail in the following chapters.

## 3. Diamond-Based Diodes

While diamond growth and doping technologies have matured in recent years, their application in diamond diodes presents unique challenges. N-type doping, crucial for forming a traditional p-n junction, remains more difficult to achieve in diamond compared to p-type doping. Consequently, the current diodes are mostly p-type Schottky diodes obtained using the gold half-contact theory, and a limited number of bipolar diodes which require successful n-type doping. This subsection reviews the development of diamond diodes from metal-diamond contacts, device structures, and so forth.

### 3.1. Schottky and Ohmic Contact

**Figure 8:**
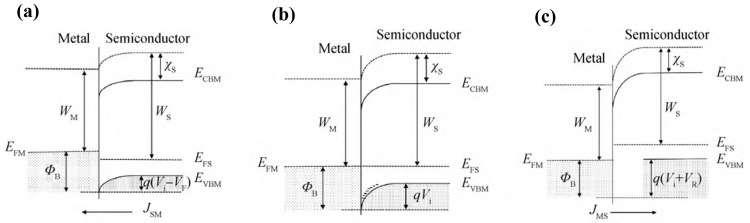
Formation of Schottky contact between metal and p-type semiconductor at (**a**) forward bias; (**b**) zero bias; and (**c**) reverse bias [102].

Taking p-type semiconductors as an example, when the metal is in contact with the semiconductor, due to the difference in Fermi energy levels, holes will be transferred between the metal and the semiconductor. If the metal Fermi energy level is higher than the semiconductor Fermi energy level, the holes in the semiconductor species will flow to the metal, resulting in the accumulation of electrons on the surface of the semiconductor. When equilibrium is reached, a built-in electric field is formed, pointing from the surface of the semiconductor. The energy band bends downwards, and the Fermi energy levels on both sides overlap, at which point the holes in the semiconductor are blocked by the surface potential barrier and can no longer diffuse into the metal. The space charge region on the surface is known as the barrier layer [31]. The barrier height can be regulated by applying a voltage to the metal electrode: by applying a positive voltage, the electric field direction is the same as the built-in electric field, which raises the barrier and further restricts the flow of holes; by applying a negative voltage to the metal electrode, the barrier decreases and breaks the equilibrium, triggering a positive current from the semiconductor to the metal, a property known as the rectification property. The equilibrium and bias process is shown in Figure 8 [102].

For an ideal gold half contact, the barrier height on the semiconductor side is:

(2) $$qV_{D}=-qV_{s}=W_{m}-W_{S}$$

The height of the potential barrier on the metal side is:

(3) $$q\varphi_{ns}=W_{m}-\chi$$

where *W_m_* is the metal work function, χ is the electron affinity energy, indicating the minimum energy required for electrons at the bottom of the semiconductor conduction band to escape from the body, and *W_s_* is the semiconductor work function, which is the disparity between the vacuum energy level and the Fermi energy level.

The Ohmic contact is the opposite of the Schottky contact. When the metal Fermi energy level is lower than the semiconductor Fermi energy level, the equilibrium formation of the semiconductor surface has a hole build-up, known as the anti-resistive layer, which is a high-conductivity region. The effect on contact resistance is very low, and there is no rectification characteristic.

Schottky diodes can be fabricated using these two contact characteristics. A Schottky diode is a type of semiconductor device that uses a metal contact to achieve similar functionality as a p-n junction diode. Unlike p-n junctions, Schottky diodes only rely on one type of charge carrier (unipolar). They achieve their forward and reverse switching characteristics through a Schottky contact on one end and an Ohmic contact on the other. A good Schottky junction is the key to achieving a high-quality Schottky diode. To obtain Schottky contact interfaces with high barriers and good adhesion and thermal stability, a variety of device and process optimizations have been performed and reported.

**Figure 9:**
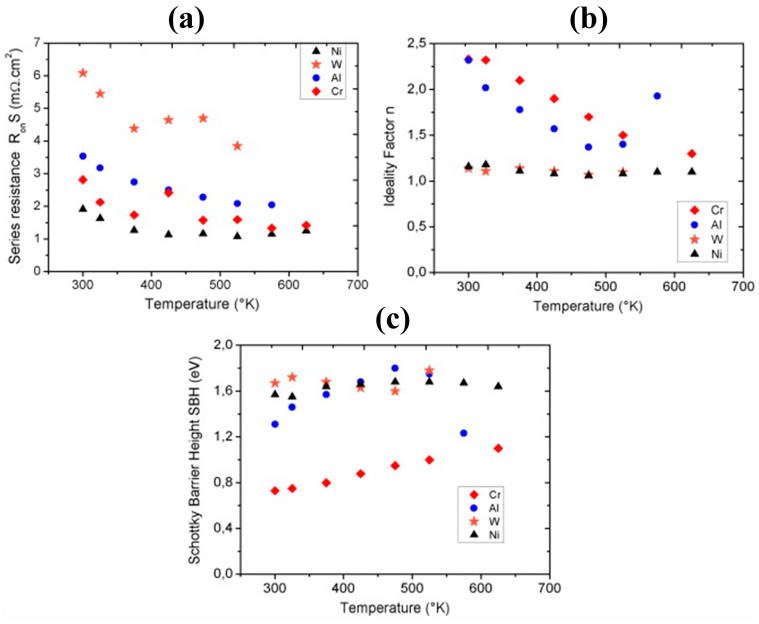
(**a**) R_on_; (**b**) ideal factor; and (**c**) SBH for W, Al, Cr, and Ni at different temperatures [103].

Koné et al. [103] compared the thermal stability of four metals, W, Al, Ni, and Cr, at the contact interface with diamond. The results show that the contact resistance of W is larger and that of Ni is smaller, as shown in Figure 9a. However, the ideal factor of W and Ni contact is more stable and near 1, and the ideal factor of Cr and Al is >1, as shown in Figure 9b. The ideal factor of W and Ni contact is more stable, and the contact properties of Cr and Al change more significantly with temperature, as shown in Figure 9c. This is because the tunneling process and the generation–complexation process can occur through the Schottky contact [104], and the combination of these parasitic phenomena with the interfacial trap density leads to a deterioration of the Schottky contact properties. It is further noted that Cr electrodes have the best adhesion to diamond.

**Figure 10:**
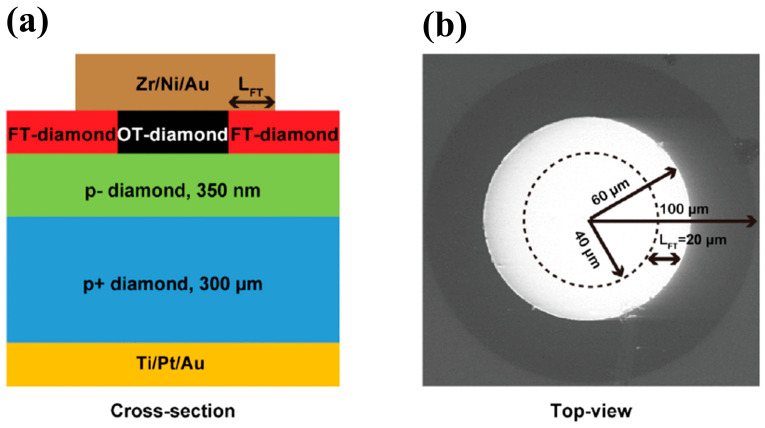
(**a**) Device schematic of the vertical diamond SBDs with FT structure. (**b**) Top-view SEM image of the SBDs [108].

Another metal, Zr, has superior properties, and diodes with Zr as the Schottky electrode maintain an ideal factor of 1.16 after annealing at 700 K [105]. The stable Schottky contact of Zr with the oxygen-terminated diamond is due to the formation of a very thin and homogeneous oxide interlayer at the interface. A Zr/Pt/Au triple metal layer is usually utilized as the electrode to improve the device’s performance and stability. Traoré et al. [106] introduced a diamond Schottky diode using Zr/Pt/Au, which achieved a breakdown field of 7.7 MV/cm and a power eutectic value of 244 MW/cm^2^. In addition, the device was thermally stable, with rectification characteristics remaining at 773 K. Oxygen-terminated and fluorine-terminated diamond surfaces have positive electron affinity potentials, which can be used to improve the Schottky contact, and have been used in the preparation of vertical Schottky diodes. Schottky contact barriers of 2 eV and 2.39 eV have been obtained, respectively [107]. Further combination and regulation of the two types of terminals have been studied to obtain a Schottky diode with improved performance (Figure 10), which has a turn-on voltage of 1.6 V, a breakdown field of 3.3 MV/cm, and R_on_ of 50.2 mΩ·cm^2^ [108].

There are two main approaches for creating Ohmic contacts in diamond [102]: one is to realize diamond contacting with the heavily doped substrate. This utilizes the tunneling effect to make the tunneling current dominant with reduced contact resistance and form an Ohmic contact. This has to be weighed against the issue of decreasing mobility brought about by the rise in the dopant concentration. The other method uses the reaction between metal and diamond at the interface to form a carbide to lower the contact barrier [109].

Many research teams have conducted a detailed study of the second method. For example, Hoff et al. [110] found that the Ti/Pt/Au contact presents rectification characteristics when annealed below 800 °C, while Ohmic contact characteristics were observed by using higher annealing temperatures above 800 °C. Kono et al. [111] calculated the barrier height between metal Ti and p-type diamond which is about −0.63 eV. This can form a good Ohmic contact, and it was also confirmed by X-ray photoelectron spectroscopy (XPS). Their XPS analyses indicate that the formation of an Ohmic contact between Ti and diamond is due to the reaction between the two at the interface, forming titanium carbide. To prevent the metal Ti from contaminating the surface, the Ti layer is often covered with a layer of Au to provide protection. However, high-temperature annealing causes mutual diffusion between Ti and Au, which increases the contact resistance and impairs the Ohmic contact. Therefore, the Ohmic contact metal widely used for diamond nowadays is a Ti/Pt/Au three-layer structure [108,112,113,114], and the Pt layer acts as a barrier layer, effectively preventing the mutual diffusion of both Ti and Au during the annealing process. In addition to Ti contact, D. Zhao et al. [115] found that metal W on p-type diamond can form an Ohmic contact after high-temperature annealing above 400 °C. F. Li et al. [116] investigated the characteristics of the contact between Au and different terminated diamonds, and the barrier height of the contact with the H-terminated diamond was −0.19 eV, which showed an Ohmic property. The Ohmic contact of Au with diamond [117] is widely used in the contact between the electrodes of the H-terminated diamond FETs, which will be discussed in the next chapter. Typical barrier heights formed by some common metals in contact with diamonds are shown in Table 2.

### 3.2. Device Structure of Diamond Diodes

**Figure 11:**
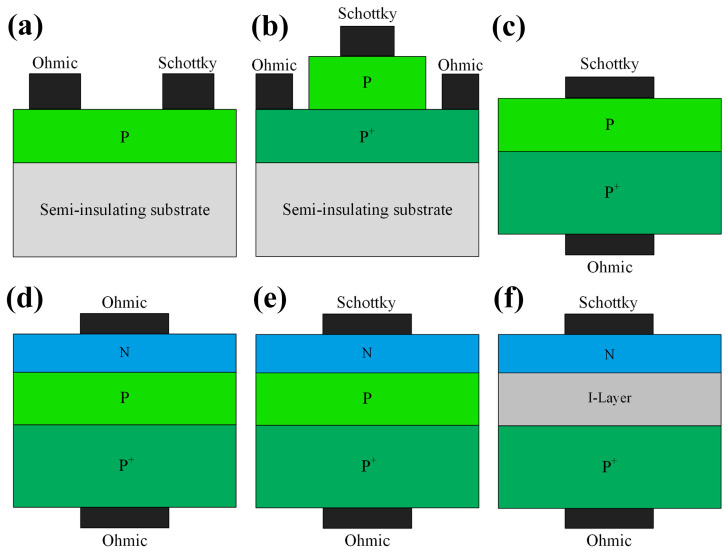
Device schematic of different diamond diodes: (**a**) LSBDs; (**b**) pVSBDs; (**c**) VSBDs; (**d**) PNDs; (**e**) SPNDs; and (**f**) SPINDs.

Diamond diodes can be divided into unipolar and bipolar types. Unipolar types are mainly lateral Schottky barrier diodes (LSBDs), pseudo-vertical Schottky barrier diodes (pVSBDs), and vertical Schottky barrier diodes (VSBDs). Bipolar types include p-i (intrinsic) -n diodes (PINDs), Schottky p-n diodes (SPNDs), and Schottky p-i-n diodes (SPINDs), as shown in Figure 11.

#### 3.2.1. VSBDs

**Figure 12:**
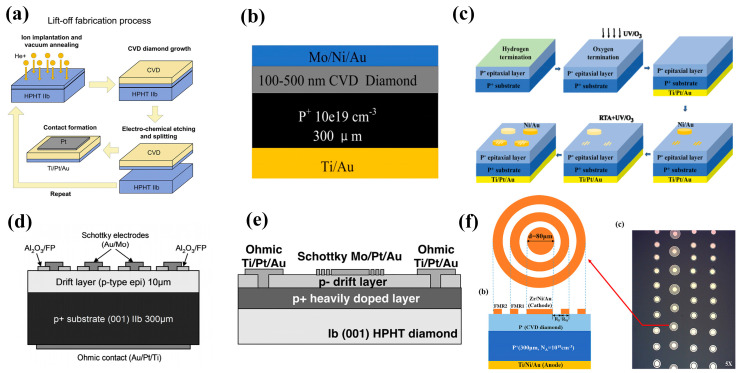
Device schematic of the diamond VSBDs with (**a**) ion implantation and two epitaxial processes [122]. (**b**) Mo/Ni/Au triple metal for Schottky contacts [123]. (**c**) Dual barriers [124]. (**d**) Al_2_O_3_ field plate [126]. (**e**) Floating field limit rings [127]. (**f**) Dual field limiting rings [128].

Vertical diamond Schottky barrier diodes (VSBDs) are widely used due to their simple fabrication process. MPCVD is usually used to epitaxially grow a lightly doped layer (p^−^) or an intrinsic layer (i) on a heavily doped substrate (p^+^). Ohmic contacts are formed on the surface of the p^+^ layer and Schottky contacts are deposited on the surface of the p^−^/i layer. The advantages are uniform electric field distribution and good conductivity, possessing thicker drift layers, and enabling high voltages and currents. In 2010, P. N. Voipe et al. [120] fabricated Schottky diodes on diamond layers doped with B. These devices demonstrated breakdown voltages over 7.5 kV and electric field ranges of 7–9.5 MV/cm at the center of the Schottky contact. In the same year, the same team reported diamond diodes with Schottky contact electrodes with a diameter of 150 μm on an oxygen-terminated diamond surface [121]. High breakdown voltages up to 10 kV and a maximum breakdown field strength of 7 MV/cm have been achieved. In 2017, Bormashov et al. [122] introduced a perpendicular Schottky diode with forward currents of up to 20 A. It utilizes Ti and Pt contacts, with R_on_ of 6 mΩ·cm^2^ and 3 mΩ·cm^2^ at room temperature and 200 °C, respectively. The study also utilized ion implantation to form an embedding layer to avoid defects contained in the substrate formed by the HPHT method from affecting the epitaxial layer, and two-step CVD to generate a bilayered epitaxial layer to enhance the mechanical strength of the film. Because the doping concentration of the epitaxial layer generated in the first step is an order of magnitude higher compared to that of the pressure-resistant region, it has a significant impact on the device in the reverse direction. Suppose the order of magnitude is higher than the withstanding region, the device’s reverse breakdown voltage and specific on-resistance will not be significantly affected (its structure is shown in Figure 12a) [122].

The metal-insulator perpendicular diode (MIPD) is an improved structure of VSBD. The mechanism is that the holes injected into the p^+^ layer have high mobility in the intrinsic layer under forward bias conditions, and the intrinsic layer blocks the high voltage under reverse bias conditions. Wang et al. [123] from Xi’an Jiaotong University used a Mo/Ni/Au three-layer metal for the Schottky contact and a Ti/Au for the Ohmic contact to prepare MIPDs with a forward current density of 7570 A/cm^2^, breakdown electric field strength of 4.2 MV/cm, and rectification ratio as high as 10^12^. The structure is shown in Figure 12b. Some common structures used for power devices have also been introduced into diamond diode fabrication. For example, Wang et al. [124] introduced a double-barrier VSBD. The double-barrier diode [125] is characterized by the use of a low-barrier Schottky contact, which can generate a forward current path to achieve a lower forward conduction voltage drop. The use of a high-barrier Schottky contact enables the depletion region generation when the diode is reverse-biased. The low-barrier region is shielded to avoid breakdown and achieve a lower reverse leakage current. This approach helps to balance the on-state voltage drop and reverse leakage current for better diode performance. In this study, Ni/Au narrow strips were first deposited on the surface of the oxygen terminal of the diamond and then subjected to RTA treatment to form a low barrier contact as an Ohmic electrode. Then, UV/O_3_ treatment was used to compensate for the partial desorption of oxygen atoms. Finally, Ni/Au was deposited once again directly as a Schottky electrode. By adjusting the proportion of narrow strips, the proportion of double barriers can be flexibly controlled, resulting in the best compromise between BV and leakage current in the device. Reducing the stripe size to be smaller than the depletion zone width is beneficial for reducing leakage current. Current research shows that the stripe width is much wider than the depletion zone width, which is not conducive to complete shielding of low barrier regions. The process and structural principles are shown in Figure 12c [124]. H. Umezawa et al. [126] enhanced the diamond VSBD’s breakdown voltage from 900 V to 1800 V with the help of the Al_2_O_3_ field plate structure, which is shown in Figure 12d. K. Driche et al. [127] improved the electric field distribution by using a floating field limited ring (FMGR) as the edge termination. The device, with a 300 nm ring spacing, had a breakdown voltage greater than 250 V and a maximum breakdown electric field of up to 2.6 MV/cm. The structure is shown in Figure 12e. Wang et al. [128] introduced two field-limited ring structures on the surface of the oxygen-terminated diamond, as shown in Figure 12f. It improved the breakdown voltage by 19% compared to the device without fabricated FMGR.

#### 3.2.2. PVSBDs/TMBS

**Figure 13:**
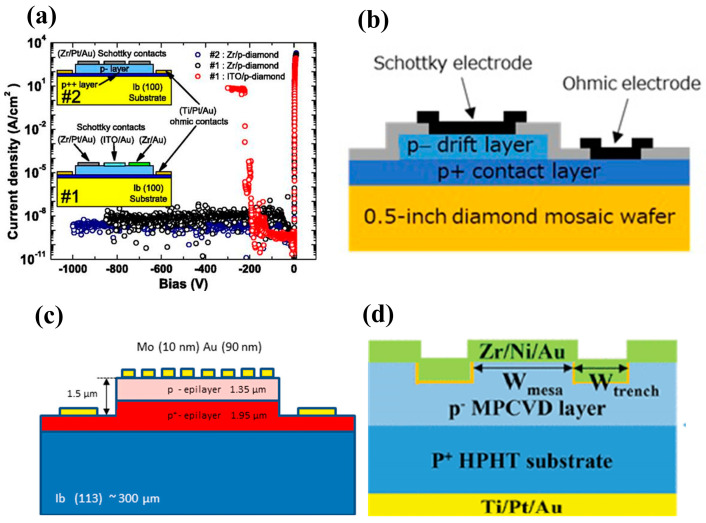
Device schematic of (**a**) pVSBDs with p^+^ doping concentration of 10^20^ cm^−3^ and p- doping concentration of 10^15^ cm^−3^ [106]; (**b**) pVSBDs using a half-inch diamond wafer [131]; (**c**) pVSBDs on (113)-oriented homogeneous epitaxial boron-doped diamond substrates [130]; and (**d**) trench MOS barrier Schottky diodes [77].

Pseudo-vertical diamond Schottky diodes (pVSBDs) [129,130] can use a lower-cost insulating substrate. The device uses an undoped substrate to produce heavily and lightly doped layers. The surface is etched below the heavily doped layer, and Schottky and Ohmic contacts are formed on the two layers, respectively. The structure is schematically shown in Figure 11b. A high current density can be achieved over a smaller area. Traore et al. [106] deposited a 200 nm p^+^ layer and a 1.3 μm p^−^ layer on a diamond substrate with a boron-doped concentration of 10^20^ cm^−3^. The concentration of the p^−^ layer was 1.5 × 10^15^ cm^−3^. This substrate was then etched to prepare a pseudo-vertical diamond diode. The structure and I-V curves are shown in Figure 13a. The breakdown field of this device is 7.7 MV/cm, and the forward current density at 6 V is 1000 A/cm^2^. H. Takanori et al. [131] fabricated pVSBDs on a half-inch single-crystal diamond wafer, as shown in Figure 13b. A total of 98% of the fabricated devices exhibited a rectification ratio better than 10^10^ and an undetectable leakage current at 5 V reverse bias. P. Hazdra et al. [130] first reported the properties of pVSBDs introduced on a (113)-oriented diamond, as shown in Figure 13c. At 180 °C, the forward current density exceeds 1 kA/cm^2^, while the reverse current density is below 10^−8^ A/cm^2^. The ideal factor of the diode is 1.23, and the Schottky barrier height is 1.71 eV. These properties are comparable to diodes introduced on (100)-oriented diamonds, and they reported that the (113)-oriented diamond has excellent surface morphology and low surface roughness [132]. This extends the substrate material with the selection range and is important for the development of diamond-based electronic devices.

The etching process in pseudo-vertical structures is also used to fabricate trench MOS barrier Schottky diodes (TMBS) [133]. This structure forms trenches on the semiconductor surface and areas covered by an oxide-insulating layer between the trenches. This allows a more uniform distribution of the electric field in the trench sidewalls and table area, thus increasing the breakdown voltage level. It has been applied in SiC [134,135], GaN [136], and Ga_2_O_3_ [137,138] power devices, and this structure in diamond was first reported in 2022. Wang et al. [77] introduced TMBS by ICP trench etching and atomic layer deposition (ALD) of alumina, as shown in Figure 13d. The following is the detailed process flow of the device: A 1 μm thick p^−^ layer was epitaxially grown on a p^+^ substrate using MPCVD and formed an oxygen terminal through UV/O_3_ treatment to achieve better Schottky contact. Ti/Pt/Au three-layer metals were deposited by electron beam evaporation (EBE) on the substrate, and Ohmic contacts were formed by rapid thermal annealing in an argon atmosphere. Then, Al was used as the mask and O_2_ was used as the etching source for ICP etching to form grooves of about 200 nm. Finally, Al_2_O_3_ was deposited by ALD, and Zr/Ni/Au three-layer metals were deposited to form Schottky contacts. The experiment achieved the highest BV of 265V with a groove ratio of 62.6%, but this resulted in a decrease in the current conduction area, which corresponded to the highest R_on_ of 5.6 mΩ·cm^2^. However, in this case, BFOM is still the highest, indicating that the optimization effect of this structure on breakdown voltage is more significant. The reverse breakdown voltage of the TMBS diode with a mesa width (W_mesa_) of 2 μm reached 265 V, 54% higher than that of the conventional Schottky diode without the trench MOS structure. Wang et al. [139] systematically simulated and investigated the effects of the trench parameters (including the dielectric layer thickness T_ox_, the mesa width W_mesa_, the etching depth D_tr,_ and the dielectric layer material type on TMBS and obtained the conclusion that relatively larger W_mesa_ and smaller D_tr_ are favorable to achieve high power merit values.

#### 3.2.3. LSBDs

Vertical-type diode devices require thicker drift layers [140,141] when considering high-voltage applications, which is a challenge for diamond growth. Pseudo-vertical diamond Schottky diodes rely on the etching process, and the flatness and uniformity after etching are unsatisfactory. Lateral-type diamond Schottky barrier diodes (LSBDs) are used to achieve high-power applications by expanding the thickness of the drift region laterally. Teraji et al. [140] introduced LSBDs using Ti/Au as a Schottky contact metal and Al as an Ohmic contact metal. The forward current density of the diode was measured to be about 0.1 A/cm^2^ at 5 V, and the breakdown field was in the range of 1.08~1.46 MV/cm. LSBDs fabricated by Z. R. Han et al. [142] achieved reverse breakdown voltages of 1159 V and 4612 V without and with field plates, respectively, with a peak current density of 5.39 mA/mm.

#### 3.2.4. PNDs/SPNDs/SPINDs

**Figure 14:**
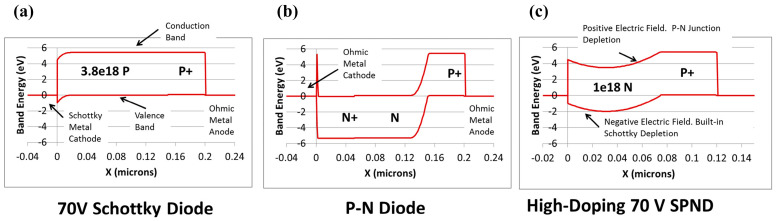
Schematic energy band diagrams of (**a**) SBDs; (**b**) PNDs; and (**c**) SPNDs [145].

The conventional pn junction diode (PND) structure in diamond is shown in Figure 11d, which is limited by the deep donor level of phosphorus, high built-in voltage and on-resistance, and long reverse recovery time [143]. It is known that the SBD is a unipolar device with the advantages of low forward conduction voltage and fast switching speed. Kubovic et al. [144] introduced a Schottky contact based on PNDs. Schottky p-n diodes (SPNDs) inherit the low forward conduction voltage characteristics of SBD while leveraging the advantages of the low reverse leakage current and high breakdown voltage afforded by the PND. The energy-band structures of the three diodes are shown in Figure 14 [145], which visually reflects the characteristics of the devices, especially the lower Schottky barrier of the SBDs and the high bandgap that the PNDs need to cross when they are turned on.

**Figure 15:**
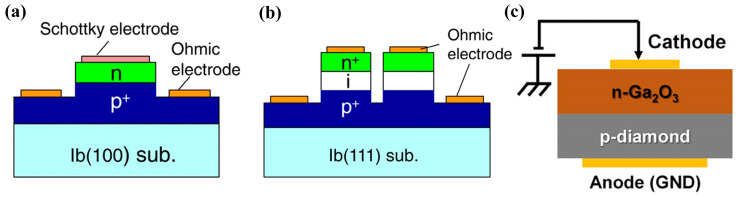
Device schematic of (**a**) SPNDs with tabletop structure and (**b**) SPINDs with tabletop structure [149]. (**c**) Diamond and Ga_2_O_3_ heterojunction p-n diodes [154].

The intrinsic drift layer of the SPINDs enables a higher breakdown voltage value than the SPNDs, but the high built-in voltage also brings about a higher on-state voltage drop and slower switching speeds [146,147]. In 2009, T. Makino et al. [148] first fabricated SPNDs with enhanced rectification characteristics. In 2014, T. Makino et al. [149] fabricated heavily doped p^+^ and n^+^ diamond films on (100) HPHT diamond substrates and (111) HPHT diamond substrates, respectively. They fabricated SPNDs and SPINDs with a tabletop structure by conventional lithography and ICP processes, in which the p-n diode has a p^+^ layer thickness of more than 5 μm and a doping concentration of ≥5 × 10^20^ cm^−3^, and the n^+^ layer’s thickness is 160 nm with a doping concentration ranging from 3 × 10^16^ cm^−3^ to 2 × 10^17^ cm^−3^ (shown in Figure 15a). The device has a maximum forward conduction current density of 60 kA/cm^2^, a switching ratio of 10^12^ at ±6 V, a reverse blocking voltage of 55 V, corresponding to a breakdown field of 3.4 MV/cm, and an R_on_ as low as 0.03 mΩ·cm^2^. The p^+^ layer of the p^+^-i-n^+^ diode has a thickness of 3 μm and a doping concentration of greater than 2 × 10^20^ cm^−3^. The i layer thickness is 200 nm, and the n^+^ layer thickness is 180 nm with a doping concentration of 1 × 10^20^ cm^−3^, as shown in Figure 15b. The R_on_ of the device is 1.4 mΩ·cm^2^, and the reverse breakdown voltage and the corresponding field strength are 92.5 V and 4.6 MV/cm, respectively. The current density is 10,000 A·cm^−2^ at room temperature when a forward bias voltage of 30 V is applied. The rectification ratio is about 10^7^. The difference in the performances of the two devices is consistent with the above analysis.

Diamond bipolar devices rely on n-type doping and after the early implementation of n-type doping on the (111) and (001) surfaces by PH_3_, research on pn bipolar diodes has been widely carried out. Due to the large activation energy required to activate the deep energy level of n-type diamond impurity P, the related devices typically operate at 200~300 °C [150,151]. The voltage difference due to the built-in electric field of the pn junction also leads to a high pass-state voltage drop [152]. Diodes with better performance are obtained largely by heterogeneous growth of gallium oxide (β-Ga_2_O_3_) on diamond substrates. In contrast to diamond, Ga_2_O_3_ does not readily form p-type doping, while it is easy to achieve n-type doping. Matsumae et al. [153] investigated a direct bonding process for the two materials, and in the following year, Sittimart et al. [154] fabricated a heterojunction bipolar diode, as shown in Figure 15c. It exhibited a rectification ratio greater than 10^8^ and a leakage current less than 10^−12^ A at ±10 V. However, the researchers also found that contact of diamond with gallium oxide may lead to tunneling currents. Thus, the failure of the rectification characteristics and the performance of diamond bipolar diodes, in general, needs urgent improvement.

In summary, researchers have explored widely on the approaches to improve the electrical performance of diamond diodes from various aspects, such as substrate selection, device structure, and heterogeneous bonding, with particular attention to the breakdown voltage, on-current, and switching ratio. Some representative diode parameters are listed in Table 3.

## 4. Diamond Field-Effect Transistors

Since the last century, researchers have been engaged in the study of diamond switching devices, such as MESFETs, MOSFETs, and other devices. As mentioned earlier, the high activation energy required for B and N doping in diamond leads to a low concentration of mobile charge carriers at room temperature. This limits the ability of the device to conduct electricity effectively. In addition, diamond surfaces naturally have a large number of surface states caused by oxygen terminals. These states act like traps for charge carriers, essentially rendering the applied electric field less effective in controlling the flow of carriers within the channel of the device, which is crucial for FET operation. Therefore, conventional diamond FETs fabricated using existing processes have not yet achieved optimal performance for switching applications. Current diamond FETs are primarily unipolar devices. Their channels are either formed by surface terminal techniques (e.g., formation of 2DHG) or by doping. While some bipolar FETs achieved through ion implantation exist, they are less common. This section delves into the basic structures of these existing diamond FETs and explores their potential applications in power electronics.

### 4.1. 2DHG

**Figure 16:**
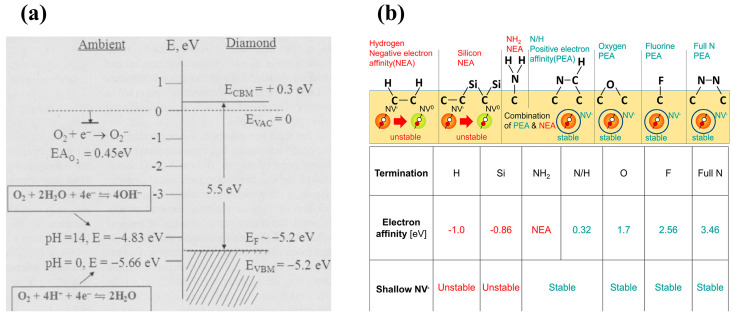
(**a**) Schematic energy band diagrams of hydrogen-terminated diamond with water adsorption layer [162]. (**b**) Chemical bond composition and electronic affinity of different terminal surfaces [93].

Hydrogen plasma treatment of the diamond surface using MPCVD technology can induce 2DHG on the diamond surface. The most widely used model is based on transfer doping, where air adsorbates behave as a host-accepted energy level on the H-terminated diamond surface. This enables the electrons inside the diamond to be efficiently transferred to the adsorbates. At the same time, a 2DHG conductive layer is formed inside the diamond [92]. The 2DHG is related to the atmospheric pH. The airborne carbon dioxide adheres to the surface water layer at pH = 6 with a potential of the water layer of about −5.3 eV, as if a p-type semiconductor forms an anti-blocking layer when it is in contact with a metal. The flow of electrons at the surface results in the formation of a region with a high concentration of holes, as shown in Figure 16a [162]. As mentioned in Section 2, for diamond, the greater the electron affinity energy of the surface terminals, the stronger the ability to attract electrons and the more difficult it is for electrons to escape from the diamond surface. Hydrogen and silicon terminals have negative electron affinity (NEA). In contrast, oxygen, fluorine, and nitrogen terminals have positive electron affinity (PEA), so the surface electrons of hydrogen terminals are easy to escape and transfer to adsorbates or dielectric layers, thus inducing 2DHG, as shown in Figure 16b [93].

In contrast, hydrogen terminals based on atmospheric transfer doping are susceptible to degradation. The surface adsorbates can be easily detached at high temperatures, and passivation layers are generally required to protect the hydrogen terminals. Gases (such as NO_2_) [163,164], molecules (such as C_60_ [165]), and transition metal oxides [166,167] with electron affinity energies more significant than the surface work function of hydrogen terminals can be used as materials for transfer doping of hydrogen terminal surfaces.

### 4.2. 2DHG FETs—Development and Optimization

Currently, available diamond FETs are mainly 2DHG devices prepared on the basis of terminal technologies such as hydrogen terminals, and this section reviews the development of 2DHGs, focusing on some schemes to optimize BV and R_on_.

**Figure 17:**
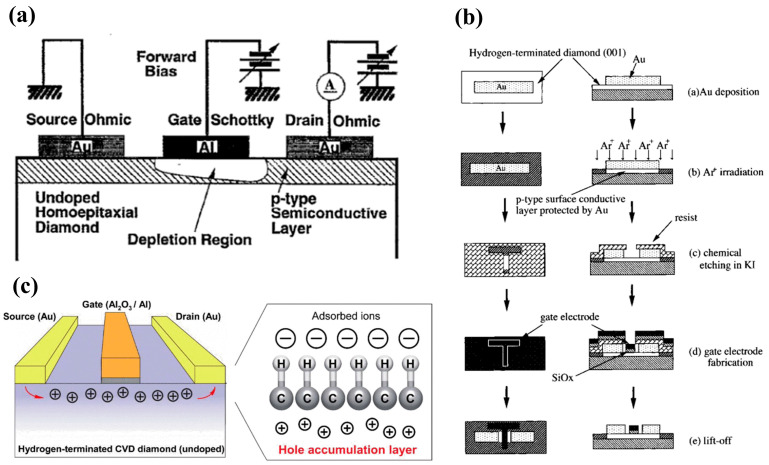
(**a**) First hydrogen-terminated normally-off MESFET devices [168]. (**b**) Process Flow of hydrogen terminal MOSFETs [171]. (**c**) Hydrogen terminal MOSFETs with an Al_2_O_3_ gate dielectric layer [173].

In the 1990s, H. Kawarada et al. [168] at Waseda University first reported a MESFET based on a H-terminated diamond, as shown in Figure 17a. The researchers used an undoped homogeneous epitaxial diamond layer as a substrate. They utilized a H-terminated surface for p-type conductivity, using Al as the Schottky gate contact and gold as the source-drain Ohmic contact. The experimental results show that the drain current is limited when V_GS_ is 0 V and does not increase until V_GS_ reaches −2 V. This demonstrates the first-ever fabrication of normally-off MESFETs on the diamond. The article also predicts that the stability of the C-H bond would begin to decrease at 300 °C in air but at up to 800 °C in ultra-high vacuum (UHV). Employing materials entirely resistant to oxidation and featuring high thermal stability could enable the device to function effectively at temperatures exceeding 500 °C.

In 1997, P. Gluche et al. [169] fabricated enhanced diamond field-effect transistors with breakdown voltages between the gate and drain exceeding 200 V. When the gate length is reduced from 8.5 µm to 3.0 µm, the drain current significantly increases from 22 mA/mm to 90 mA/mm. This trend suggests that further miniaturization, potentially below 1 µm, could lead to even higher current capabilities. Notably, if the breakdown voltage remains stable under these conditions, the radio-frequency (RF) power handling of the device is expected to surpass 6 W/mm. This research also allows for replacing surface hydrogen with oxygen plasma treatment, resulting in highly insulating oxygen-terminated surfaces which is an effective device isolation method widely used today. The main contribution of this research is to demonstrate the potential of diamond FETs for high breakdown voltage and high-power density applications.

Following this breakthrough, H-terminated FET research experienced rapid progress. In 1998, H. Kawarada et al. [170] introduced H-terminated diamond MESFETs using a heteroepitaxial diamond grown on a β-SiC substrate, which used Cu as the gate and Au as the source and drain. This work pointed out that heteroepitaxial diamond devices based on heteroepitaxial epitaxial diamond devices were able to achieve a similar level as homoepitaxial diamond devices. In 1999, A. Hokazono et al. [171] introduced H-terminated diamond MOSFETs using silicon oxide as the gate insulating medium. They obtained a 16 mS/mm transconductance when the gate length was 6 μm. In addition, due to the simplicity and the capability of self-alignment, the process demonstrated in this work, shown in Figure 17b, has been widely adopted for diamond-based MOSFET fabrication.

In 2004, M. Kasu et al. [172] investigated the effect of crystal quality on the conductivity of H-terminated diamonds and found that crystal defects in the epitaxial layer can lead to gate leakage current in the device. At the same time, residual B impurities in the epitaxial layer led to substrate leakage in the DC characteristics. In 2007, K. Hirama et al. [173] comprehensively reported the surface hole accumulation layer model in H-terminated diamond MOSFETs. C-V and I-V characteristics were measured to assess the electrical behavior of these devices. Microwave power characteristics were also evaluated, providing insights into their high-frequency performance. These analyses revealed that large-grain polycrystalline diamond with a preferred (110) orientation and hydrogenated surface termination exhibits a lower square resistance. The electric field distribution within this device structure is visualized in Figure 17c.

Prior to 2013, research primarily focused on establishing and evaluating the fundamental device structures. It was generally believed that air adsorbates were essential for the conductivity of diamond devices. Still, detachment from adsorption would occur at high temperatures degrading the device performance. As a result, the film deposition at the early experimental stage was carried out at low temperatures, generally below 300 °C, limiting the exploitation of the inherent advantages of diamond’s exceptional thermal conductivity. After that, the research focused on high-temperature insulation films, vertical structures, and new structures, which led to the emergence of more advanced devices for high-power applications. In 2012, A. Hiraiwa et al. [174] reported for the first time that a high-temperature ALD-Al_2_O_3_ insulating film deposited at 45 °C can maintain the conductivity of H-terminated diamond until 550 °C. They experimentally confirmed that the adsorbates on the surface of the hydrogen terminals at room temperature during ALD are detached at 450 °C, indicating the existence of other mechanisms. Detachment suggests the existence of different mechanisms capable of maintaining 2DHG.

In 2014, H. Kawarada et al. [175] developed a method using a high-temperature ALD process to create a thick and insulating Al_2_O_3_ film. This film served as a protective layer on a H-terminated diamond surface. This approach resulted in a diamond power device with the advantages of high-temperature stability and BV. The BV was close to 1 kV and the maximum breakdown field was about 3.6 MV/cm. In their subsequent studies, the relevant mechanism of Al_2_O_3_ in enhancing hydrogen terminals was studied [176]. As mentioned in the article, unoccupied energy levels in Al_2_O_3_ (such as gap oxygen defects) can trap electrons. This negative charge effect helps to maintain the electronic structure on the diamond surface of the hydrogen terminal, thus maintaining the 2DHG.

**Figure 18:**
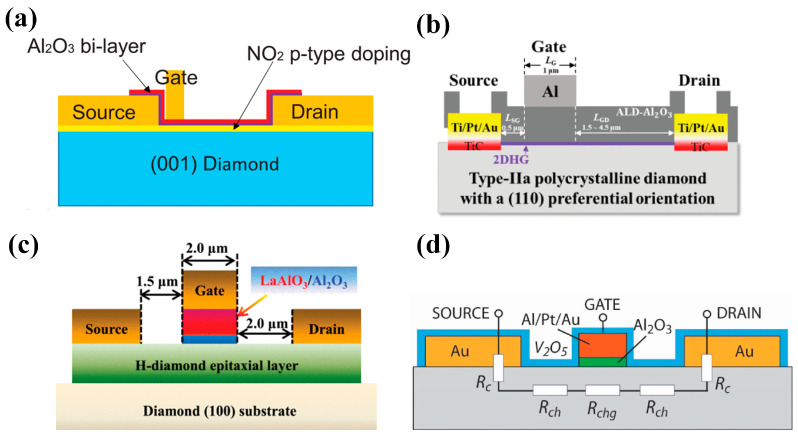
Device schematic of the diamond MOSFETs with (**a**) 16 nm Al_2_O_3_ gate dielectric layer and NO_2_ doped substrate [177]; (**b**) an asymmetric construction and Al_2_O_3_ gate dielectric layer [180]; (**c**) a LaAlO_3_ and Al_2_O_3_ gate dielectric layer [181]; and (**d**) a V_2_O_5_ and Al_2_O_3_ gate dielectric layer [182].

Diamond FET devices utilizing Al_2_O_3_ as a gate dielectric or passivation layer achieved good performance. Koyama et al. [177] deposited a 16 nm thick Al_2_O_3_ layer by ALD on a (001) diamond surface, which is doped by NO_2_, as shown in Figure 18a. A high voltage of 3659 V was achieved at an L_GD_ of 50 μm, which is the highest value of diamond MOSFETs. In this study, a BFOM with a maximum of 173 MW/cm^2^ was obtained at a gate length of 2.5 μm, corresponding to a BV and R_on_ of 1528 V and 13.48 mΩ·cm^2^, respectively. The devices with the highest BFOM (~875 MW/cm^2^) [39] obtained so far were also doped by NO_2_ and utilized Al_2_O_3_ as the dielectric layer. The device was realized on a diamond substrate obtained by high-quality heterogeneous epitaxial growth. The substrate surface was subjected to a 200 h chemical mechanical polishing process to effectively remove surface damage due to mechanical polishing, and a low-resistance diamond surface was obtained. Then a high proportion of H_2_ was introduced during the epitaxial layer growth process to form a H-terminated surface, which was then doped with NO_2_ to increase the hole concentration. The hole concentration of the device is increased by almost an order of magnitude (to about 10^14^ cm^−2^) compared to the conducting channel formed by ordinary H-termination diamond [178,179]. Then a double layer of a total of 16 nm thick Al_2_O_3_ was deposited using ALD, and these efforts resulted in a device with a BV of 2568 V and an R_on_ of 7.54 mΩ·cm^2^. Kudara et al. [180] achieved an output power density of 2.5 W/mm at 1 GHz by using an asymmetric structure of 200 nm thick ALD-Al_2_O_3_ with unequal L_GD_ and L_GS_, as shown in Figure 18b. Liu et al. [181] deposited LaAlO_3_ on the Al_2_O_3_ layer achieving enhanced-mode MOSFET, which is illustrated in Figure 18c. The device and the depletion-mode MOSFETs with a single Al_2_O_3_ layer exhibit almost the same extrinsic transconductance with a maximum value of 17 mS/mm^2^. Leveraging this performance, they successfully fabricated NOT and NOR logic circuits. These results demonstrate the promising potential of diamond MOSFETs for future applications in logic circuits. Macdonald et al. [182] improved the performance of diamond MOSFETs with a gate length of 250 nm by performing a 400 °C annealing treatment and introducing V_2_O_5_ instead of Al_2_O_3_ as a surface electron acceptor layer, as shown in Figure 18d. The maximum drain current I_D_MAX_ was increased from 100 mA/mm to 375 mA/mm after annealing, and with the introduction of V_2_O_5_, the extrinsic transconductance was increased from 49 mS/mm to 98 mS/mm. The R_on_ was reduced from 43.9 mΩ·cm^2^ to 16.8 mΩ·cm^2^.

**Figure 19:**
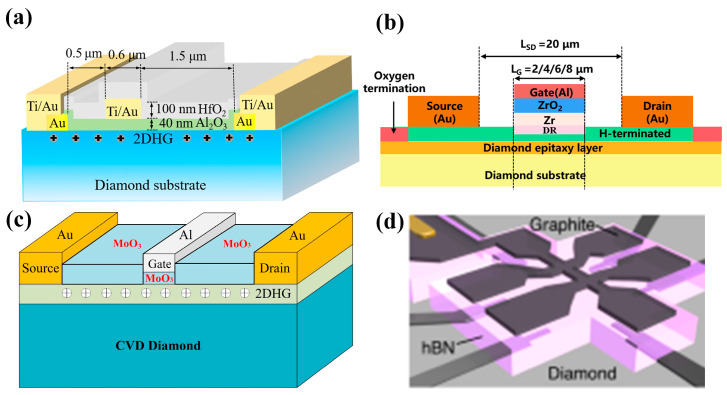
Device schematic of the diamond MOSFETs with (**a**) a HfO_2_ and Al_2_O_3_ gate dielectric layer [191]; (**b**) a ZrO_2_ dielectric layer [192]; (**c**) a MoO_3_ gate dielectric layer [187]; and (**d**) a two-dimensional material h-BN gate dielectric layer [195].

The above studies on Al_2_O_3_ dielectrics have progressively involved other dielectric materials. Research has shown that various materials can effectively maintain and improve the hydrogen termination on diamond surfaces. This metal–insulator–semiconductor (MIS) structure plays a crucial role in regulating the charge carriers within the device. Notably, several studies have explored converting depletion-mode diamond FETs to enhancement-mode by introducing specific insulating gate materials [183,184]. These dielectric materials include the metal oxide materials already mentioned in Section 4.1, such as Y_2_O_3_ [185], MoO_3_ [186,187], WO_3_ [188], HfO_2_ [189,190,191], ZrO_2_ [192], TiO_x_ [193], etc. In addition, some two-dimensional materials are also used as dielectric layers, which can reduce the effect of the interfacial roughness and promote the charge transfer at the interface between the H-diamond and the dielectric layer [194], such as h-BN (as shown in Figure 19 [195,196]).

Figure 19a [191] illustrates MOSFETs with 40 nm Al_2_O_3_ and 100 nm HfO_2_ stacked gate dielectrics, and the device successfully reduces the leakage current and achieves a high switching current ratio of 10^11^, which is the highest value in diamond FETs so far. Figure 19b demonstrates a MISFET utilizing a high-k material of ZrO_2_ (k = 15.4) as the dielectric layer [192]. This device maintained the high V_TH_ of 2.5 V without sacrificing I_D_MAX_ (about −74 mA/mm) too much. Figure 19c shows the MOSFETs using MoO_3_ [187] with k values of about 12 to 18, which obtained an R_on_ of 76.54 Ω·mm. The highest k value of the gate medium layer is 27.2 [197], which is achieved by growing stacks of TiO_2_ and Al_2_O_3_.

In 2022, Yosuke Sasama et al. [195] reported their work in Nature which was about using h-BN as the gate dielectric material and graphite as the gate for high mobility FETs. The entire device fabrication process was carried out in a high-vacuum, argon-filled glove box with an oxygen content of less than 0.5 ppm and a water content of less than 2 ppm. Such an environment can significantly reduce the influence of atmospheric receptors on the diamond surface and help to maintain the cleanliness of the diamond surface so that surface scattering is reduced and carrier mobility improved. The high dielectric constant and low loss characteristics of h-BN ensure the high-frequency performance of the diamond device. The devices exhibit high mobility of 680 cm^2^/V·s and over 1000 cm^2^/V·s at room temperature and 150 K, respectively, which provides a solid foundation for further mobility improvement of diamond FET devices.

**Figure 20:**
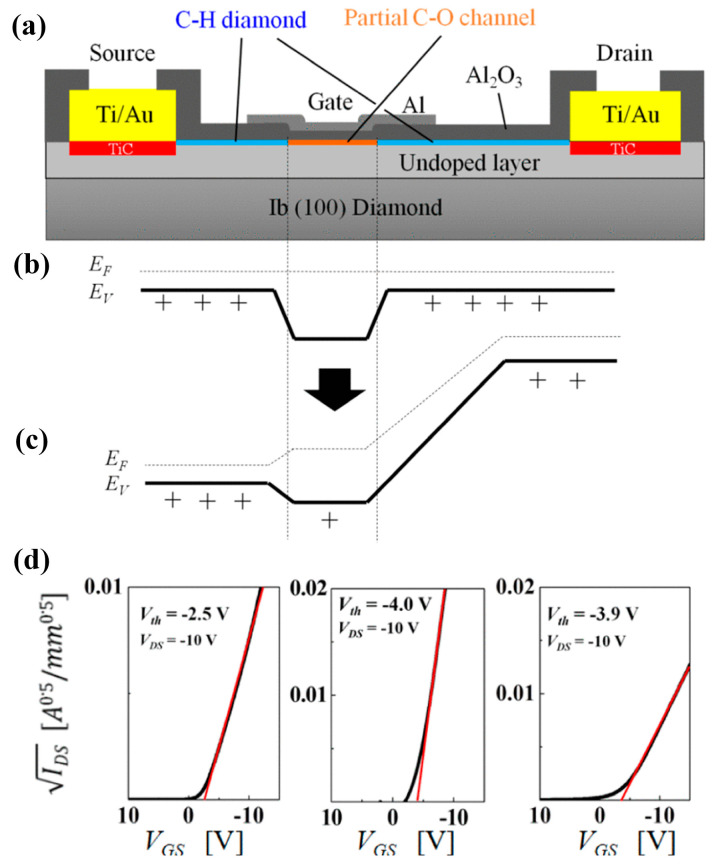
(**a**) Schematic of the structure of partial oxygen-terminal channel diamond MOSFETs. Schematic energy band diagram of (**b**) off-state and (**c**) on-state. (**d**) Transfer characteristic curves for different devices [198].

In addition to the formation of 2DHG by hydrogen termination for preparing FETs, there are related reports on oxygen, fluorine, and silicon termination. Oxygen termination is mainly used for surface modification. By partially modifying the hydrogen termination into oxygen termination, the threshold voltage of the FETs can be regulated to turn the device into an enhanced-mode, as shown in Figure 20a [198]. The oxygen termination region is obtained by etching part of the Al_2_O_3_ and then treating with UV-O_3_ for 3–5 min, which plays the role of blocking hole conduction without bias and induces positive charges on the surface by applying negative pressure at the gate before conduction. Unlike depletion-mode devices that conduct current without an applied gate voltage, these enhanced-mode devices require a positive voltage at the gate to induce conduction (as illustrated in Figure 20b,c). This behavior is reflected in the transfer curve of Figure 20d, where the threshold voltage lies between −2.5 V and −4.0 V. Notably, these devices achieved a high breakdown voltage of 2021 V.

**Figure 21:**
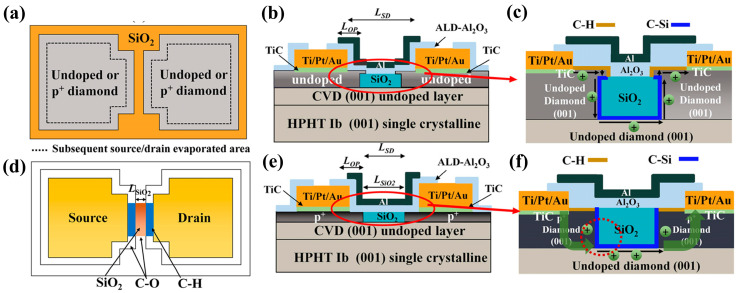
Device schematic of (**a**) the top view of the diamond silicon-terminated MOSFET; (**b**) undoped devices; (**c**) magnified image of the red circled region in (**b**); (**d**) the top view of the channel compositions; (**e**) heavily doped devices; (**f**) magnified image of the red circled region in (**e**) [200].

Compared to H-terminated diamond devices, the research on Si-terminated diamonds was initiated later. From 2019 to 2020, W. Fei et al. [199,200] reported the principle and fabrication of Si-terminated diamond FETs for the first time. During the selective epitaxial growth of a diamond using TEOS-SiO_2_ as a mask, the oxygen terminals on the diamond surface were replaced by a C-Si structure. The top view of the undoped silicon-terminated MOSFET is shown in Figure 21a, and the front view is shown in Figure 21b. The presence of C-Si bonds under the SiO_2_ mask region was confirmed by transmission electron microscopy (TEM) and energy dispersive X-ray spectroscopy (EDS). Figure 21c shows the magnified image of the red circled region in Figure 21b, the hole current passes through the vertical diamond and the sidewall faced to the SiO_2_. Figure 21d gives the various components of the device in the top-view perspective. The undoped device demonstrates a lower I_D_MAX_ of about 17 mA/mm and a V_TH_ of −19V, while the heavily doped device, as shown in Figure 21e, demonstrates a higher I_D_MAX_ of 165 mA/mm with a V_TH_ of −6 V. Figure 21f shows the magnified image of the red circled region in Figure 21e, compared to undoped devices, the hole channel increases and the S/D resistance is negligible.

Si-terminated diamond can directly form an interface with SiO_2_, which is the most commonly used gate insulator in Si-based MOS devices with very high reliability and stability [201]. The interfacial density of states (D_it_) at the C-Si/SiO_2_ interface is comparable to that of H-terminated diamond surfaces [202,203], showing versatile potential applications of the different terminal techniques of diamond.

Compared with H-terminated devices, Si-terminated devices have shown higher threshold voltage and hole mobility [204]. Also, they have better stability [100] due to more dangling bonds on the surface of C-Si as compared to C-H [205,206]. While Si-terminated diamond FETs offer promise, some studies have identified an issue with variations in the threshold voltage across different devices. This non-uniform V_th_ distribution needs to be addressed for consistent device performance, and researchers have explored ways to overcome this issue. Some studies identified devices exhibiting depletion-mode behavior [207], and based on this finding, researchers introduced C-Si-O surfaces on the silicon-terminated diamonds [206,208]. This modification offered multiple benefits like enhanced negative electron affinity and easier turn-off. H. Kawarada et al. [209] fabricated C-Si-O surfaces by both SiO_2_ reduction and direct deposition methods, achieving high mobility (>150 cm^2^/V·s) and significant negative threshold voltage (V_TH_ < −3 V). Overall, the research on Si-terminated diamond MOSFETs is still in its infancy, with low current levels and insufficiently stable thresholds, and high-breakdown-voltage- and high-current-density-related devices have not yet been reported.

### 4.3. Other Structures: VMOSFETs, BJTs, JFETs, and So On

In addition to the conventional FET structure, vertical MOS field-effect transistors (VMOSFETs) [210], bipolar junction transistors (BJTs) [211,212,213], and junction field-effect transistors (JFETs) [214,215], which are common in power devices, are also applied in diamond devices.

The vertical structure is typical in power devices which can increase the length of the drift region in the vertical direction to achieve higher current density. This is beneficial to reduce the chip area, and at the same time has enhanced channel control and smaller parasitic capacitance [216,217,218].

**Figure 22:**
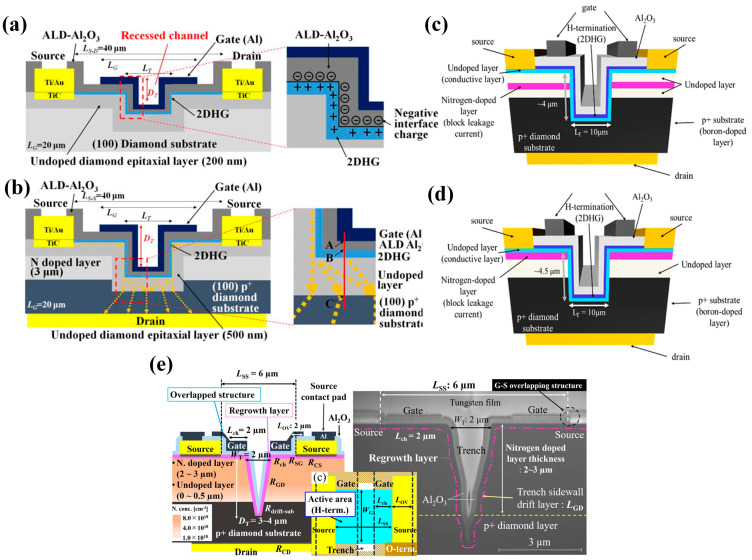
Device schematic of U-trench diamond MOSFETs with a (**a**) lateral structure; (**b**) vertical structure [219]; (**c**) barrier layer formed by ion implantation; and (**d**) epitaxy [220]. (**e**) Device schematic of vertical V-trench diamond MOSFETs with a highly concentrated N-diffusion barrier layer [221].

Vertical-type diamond MOSFETs have been experimentally studied and reported. In 2016, M. Inaba et al. [219] reported the first vertical-type H-terminated diamond devices. They introduced lateral-type and vertical-type trench structures, as shown in Figure 22a,b. The two types of devices obtained I_D_MAX_ of about −18 mA/mm and −4 mA/mm, respectively, and the switching ratio of the vertical-type MOS was about 10^4^. In 2018, S. Okubo et al. [220] introduced an Al_2_O_3_ dielectric based on the above structure. They added an n-type doping layer to reduce the leakage by injection and epitaxy, as shown in Figure 22c,d, respectively. This design increased the breakdown voltage and achieved a maximum drain current density of over 200 mA/mm, with a high switching ratio of over 10^8^. The specific on-resistance is 31 mΩ·cm^2^ and 41 mΩ·cm^2^ for the devices with an n-type implanted layer and n-type epitaxial layer, respectively. In 2020, M. Iwataki et al. [221] utilized a highly concentrated N-doped layer for leakage current suppression and introduced a device with a trench width of 2 μm, which is shown in Figure 22e. The device can maintain a high switching ratio of 10^7^ at 200 °C, with a maximum I_D_MAX_ of 12,000 A/cm^2^ at room temperature, and a specific on-resistance of 3.2 mΩ·cm^2^. These results are the optimal values for the current vertical diamond MOSFETs. The BFOM was estimated to be around 31 MW/cm^2^.

In 1982, J. F. Prins [211] first used natural type IIb (i.e., p-type) diamond as a substrate and introduced an n-type layer by the ion implantation technique to prepare BJTs. The formation of p-n junctions was observed, and diode properties were achieved. Although the current amplification factor of these BJTs was only 0.11, it proved the feasibility of the diamond transistor. T. Makino et al. [212] fabricated and tested (001) bipolar transistors (BJTs) with a vertical p-n-p structure on a crystal-oriented diamond. The device produced a current response in the range of 100 nA–50 μA at room temperature, improving the rectification characteristics of the diamond BJTs. The BV of the device is greater than 100 V at room temperature. The co-base current amplification factor α is between 0.2 and 0.45 by measuring the emitter current (I_e_) and collector current (I_c_). It is difficult for the carriers to cross the thicker base region, which is the main reason for the small amplification factor.

The difficulty of diamond n-type doping can be overcome by combining other materials with diamond to form a heterostructure. In 2020, D. Liu et al. [213] fabricated p-n-p-type AlGaAs/GaAs/diamond heterojunction bipolar transistors (HBTs), in which n^+^ doped GaAs was grafted onto a p-type diamond by bonding. The ideal factor of this p-n junction was 3.67 and an I_on_/I_off_ ratio of 3.74 × 10^10^, which exhibits good diode characteristics, suggesting that HBTs have good prospects for development. However, the current gain β of the device is about 1, which is attributed to the heterojunction barrier (about 0.3 V) preventing hole injection into the collector region. This can be expected to be overcome by tuning the electron affinity of the diamond surface.

In 1999, A. Aleksov et al. [222] first reported diamond JFETs, which have a 100 mA/mm drain current density and can be operated at 200–250 °C. In 2014, T. Iwasaki et al. [223] fabricated JFETs with a BV of 600 V and a BFOM of about 100 MW/cm^2^. In 2015, T. Iwasaki et al. [224] achieved conductive modulation by n-type compensated doping in the p-type region, which resulted in an 8.5-fold increase in I_D_ and a current gain between 100 and 2600. In 2016, T. Suwa et al. [215] designed JFETs with tapered and gradient-doped channels to realize a device of normally-off type with a threshold voltage of −3.0 V. It was shown that the threshold voltage of the device could be modulated by changing the channel width. On this basis, in the literature published by the same researcher in the coming year [225], normal-off devices are also achieved by making narrow channels (0.5 μm). The device also added the p^+^ contact layer at the source/drain and enhanced the bipolar mode of operation at temperatures of 473 K and 573 K.

**Figure 23:**
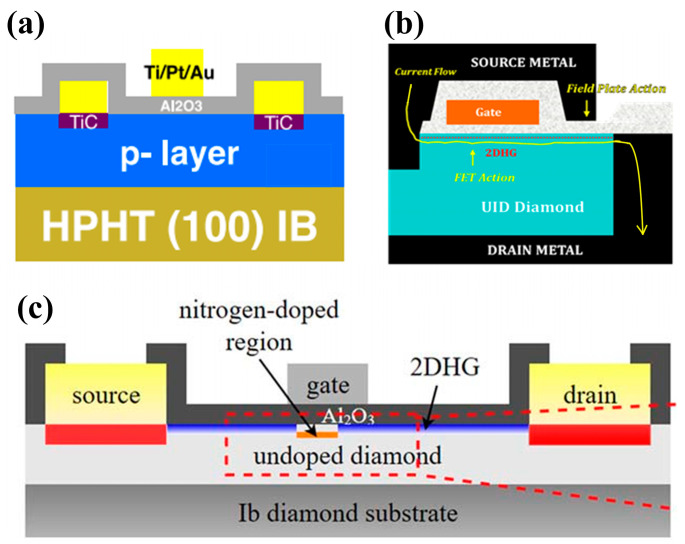
Device schematic of (**a**) deep depletion diamond MOSFETs [226]; (**b**) cross-section of a high-voltage, quasi-lateral diamond MOSFET design [227]; and (**c**) MOSFETs with nitrogen-implanted region [228].

In addition, there are some special schemes that optimize the parameters of the devices, and since there are fewer relevant reports on these structures, they are listed integrally as shown in Figure 23. T. T. Pham et al. [226] deposited a 40 nm alumina layer on the surface of an O-terminated p-type diamond with Ti/Pt/Au as the gate contact. A deep depletion-mode MOSFET is achieved with a breakdown voltage of 200 V, as shown in Figure 23a. Guo et al. [227] proposed a novel high-voltage, quasi-transverse diamond power MOSFET structure that utilized extended lateral and vertical drift regions for voltage blocking while introducing a field plate in the drift region to further improve the electric field distribution (Figure 23b). The maximum BFOM value obtained from the simulation is 6672.3 MW/cm^2^, corresponding to a BV and an R_on_ of 5.6 kV and 4.7 mΩ·cm^2^, respectively. These results have exceeded the theoretical limit values for GaN and SiC devices, demonstrating an extremely high upper limit for diamond power devices. Nobutaka Oi et al. [228] achieved the normally-off diamond MOSFETs by forming a shallow nitrogen doped layer below the C-H channel region by a nitrogen ion implantation technique. The device shows a breakdown voltage of up to 1600 V and a maximum breakdown electric field of 2.7 MV/cm, which demonstrates the potential of the ion implantation technique in the fabrication of diamond MOSFETs.

### 4.4. Summary

For undoped diamond MOSFETs, the device performance relies on the regulation of different surface terminals and dielectric layers. Research on diamond FETs has progressed significantly, particularly those utilizing 2DHG channels. Early studies focused on understanding terminal configurations and the 2DHG formation itself. Now, the technology for fabricating 2DHG-based FETs is becoming more mature, with advancements in gate dielectric optimization combined with surface terminations, as well as heterogeneous epitaxy techniques. In addition, junctions or drift layer formation by epitaxy or ion implantation has been investigated. Table 4 comprehensively compares the electrical performance of the electrical performance of various diamond FETs. The parameters are selected from the maximum values mentioned in the literature, not necessarily from the same device.

## 5. Application of Diamond in Electrical Circuits

Diamond has high carrier mobility and breakdown field strength, and diamond diodes have a high quality factor, which is expected to be advantageous in high-power electronics applications. Diamond Schottky diodes are the most mature diamond semiconductor devices, which have begun to be used in circuits, especially converters, and inverters, but the small size of the diamond substrate limits the integration of diamond semiconductors, so the current application is generally to connect multiple diodes in parallel to carry higher currents, as well as combining diamond diodes with MOSFETs prepared from other substrate materials to form staggered structures to improve thermal management and enhance dynamic performance by staggering phases to increase current capacity. A buck converter was designed using parallel diamond diodes and an interleaved structure in which the diamond is coupled with power devices made of other materials. The parallel structure effectively improves the low current situation of the diamond devices due to size constraints [233]. T/3 for each branch optimizes the effective total current distribution between the diodes and increases the power converter’s current and output signal frequency. In double-pulse tests, the maximum dV/dt of the diode was 16.4 V/ns when the diode was turned off and was 2 V/ns when it was turned on.

**Figure 24:**
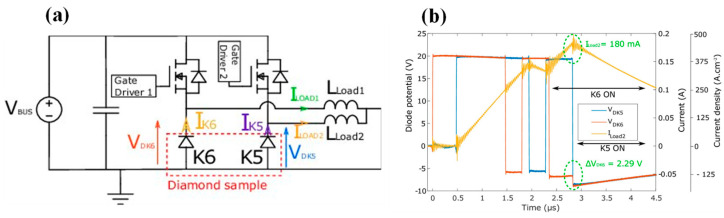
(**a**) Schematic of the interleaved buck converter in the double-pulse test configurations. (**b**) Double-pulse test of the interleaved buck converter using two legs with diamond SBDs [234].

Another study on diamond buck converters is shown below [234]. Figure 24a shows a DC-DC converter formed by two parallel diamond diodes (K5 and K6, two diamond devices with pVSBD structures) and an interleaved structure connected in series with Si MOSFETs, which is used to convert the input DC voltage to a lower DC voltage. When turned on, the MOSFET first conducts to charge the inductor, and the current flows through the MOSFET and diamond SBD; When the MOSFET is turned off, the current of the inductor continues to flow through the DSBD. At this time, the DSBD conducts, and the inductor releases energy to the load. The capacitor is usually used to filter out the ripple of the output voltage, providing a smoother DC output. However, Figure 24b shows the dynamic behavior of the interleaved buck converter in a dual-pulse test configuration, where there was a delay in the opening of both branches during the test. The curve in the figure shows the potential at the negative end of the diode and the current I_load2_ of the K5 branch. It can be seen that when K5 is turned on, some of the current is added to K6 (Iload2 decreases in the imaginary circle), which increases the state voltage drop of K6 (ΔV_DK6_ increases). This is due to the common impedance caused by the parallel structure, which is a defect of the current diamond converter. Due to the fact that parallel diamond diodes actually share a p^+^ layer, it is mentioned in the article that the thickness of the p^+^ layer can be modified, the current path can be increased, or the electrical isolation between cathodes can be achieved through RIE technology to reduce or eliminate the common resistance. This poses requirements for device manufacturing processes, and future device design and preparation should consider compatibility with applications.

**Figure 25:**
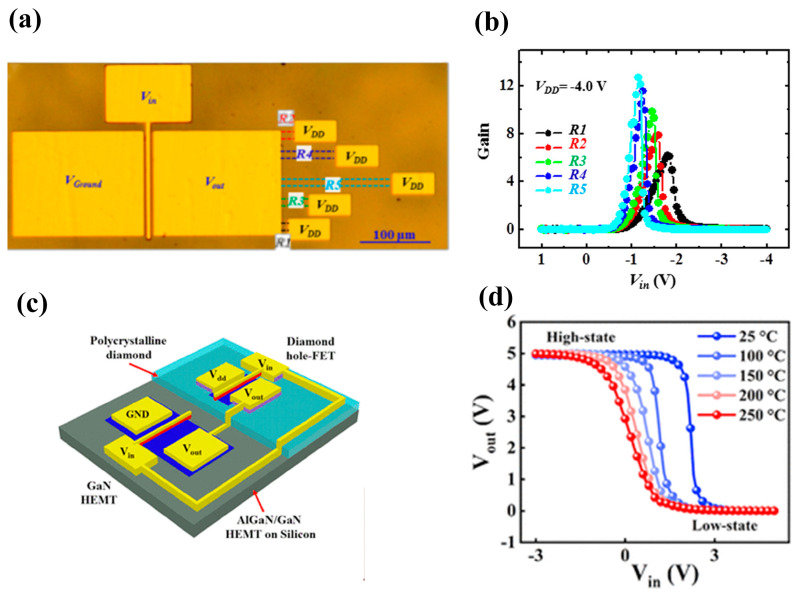
Schematic of the (**a**) diamond inverter equipment and (**b**) gain curve [197]. (**c**) Schematic of an inverter with heterogeneous integration of GaN HEMTs and diamond FETs and (**d**) VTC curves [234].

Recently, diamond MOSFETs have been used in logic circuits. In 2017, Liu et al. [197] deposited TiO_2_/Al_2_O_3_ bilayer films on a diamond substrate, which significantly improved the C-V characteristics of MOSFETs by optimizing the thickness of Al_2_O_3_ buffer layer deposited by ALD. The FET has a switch ratio of 10^9^ and a subthreshold switch of 79 mV/dec. The inverter fabricated based on the device shows prominent electrical characteristics, and the gain is between 6.2 and 12.7 under different load resistances, as shown in Figure 25a,b.

In 2021, Ren et al. [234] successfully grew a p-type H-terminated diamond epitaxially on an AlGaN/GaN high electron mobility transistor (HEMT) and further fabricated an inverter circuit, as shown in Figure 25c. The inverter has a VTC test curve at 25–250 °C, as shown in Figure 25d. The input voltage (V_in_) scans from −3 V to 5 V show a more stable operating performance at 250 °C.

**Figure 26:**
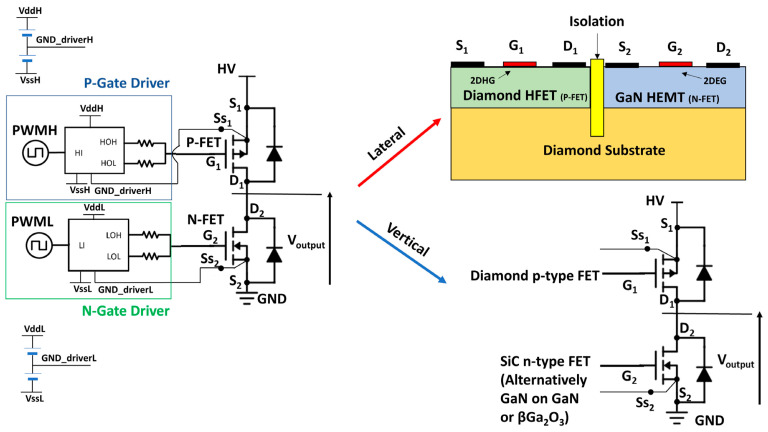
Schematic of a possible monolithic integration of diamond and other WBG/UWBG semiconductors [152].

Currently, diamond is rarely used in CMOS inverters, which is attributed to the scarcity of n-type doped diamonds. Practical applications also require n-type device research or heterogeneous integration technology. N. Donato et al. [152] similarly summarized the trend towards diamond CMOS, as shown in Figure 26, which can be achieved by heterogeneous integration of diamond and GaN on the same substrate or by combining diamond PMOS with other kinds of NMOS.

## 6. Summary and Prospect

### 6.1. Status and Challenges of Diamond Parameters, Commercialization, and Integration

This review explores the potential of diamond for next-generation electronics, particularly in high-power applications. We begin by highlighting the unique properties of diamond, including its exceptional thermal conductivity, breakdown field strength, and carrier mobility. These characteristics make diamond a highly attractive candidate for power devices compared to traditional materials like silicon.

The excellent properties of the diamond material have aroused great interest from the industry towards practical power electronics applications. However, diamond semiconductor research is still in its early stages. Table 5 gives a comparison of the electrical parameters of diamond and SiC, GaN, and β-Ga_2_O_3_ devices, with the highest BFOM as the selection criterion.

Among them, SiC and GaN have been commercialized; SiC devices are commonly used in high-power converters and inverters, such as Infineon’s CoolSiC^TM^ MOSFET series and Nexperia’s 1200 V SiC MOSFET series. GaN is commonly used for high-speed switching to achieve the lowest switching loss, such as Infineon’s CoolGaN^TM^ 600 V series. β-Ga_2_O_3_ and diamond devices are currently in the research stage. It can be seen that even though the research on diamond devices started late, its high BFOM advantage has begun to be reflected. According to the research, diamond seems to be the only semiconductor with a sharp decrease in resistivity with temperature. This is the advantage of diamond in terms of power, highlighting its importance in the field of power electronics.

However, although diamond devices have such ideal properties and have made significant breakthroughs in research, there is still a long way to go for integration with existing technologies and further commercialization. To take advantage of diamond, device performance needs to be further improved. The following is a list of the expectations for the relevant parameters of the diamond devices.

(a) BV: For diode devices, the current vertical devices generally achieve greater than 1 kV with the highest close to 10 kV applications; the future goal is to break through 10 kV without affecting the on-state current. For field-effect transistors, the current maximum breakdown voltage of 2~4 kV; the future should break through more than 10 kV.
(b) On current: The open-state current of most devices is in the range of 1~10 A, and the future goal should be to realize the application of more than 10 A. The current density of a diode is expected to break through 100 KA/cm^2^, and the field-effect tube breaks through 10 A/mm.
(c) Switching speed: The current diamond diode slew rate is less than 10 V/ns; future expectations exceed 100 V/ns.
(d) BFOM: The current BFOM values for diamond diodes and FETs are mainly in the range of 10 to 10^3^ MV/cm^2^, and ideally one should expect more than 10^4^ MV/cm^2^ at the maximum breakdown field strength close to 10 MV/cm [242].

For integration and commercial applications, the major semiconductor companies have not yet utilized diamonds for devices. 

The following are the challenges and some solutions for integrating diamond with existing technologies and achieving commercial applications:

(a) Material quality and cost control: High-quality electronic-grade diamond wafers are expensive to produce and are usually small in size (less than 1 inch). Future wafers grown by HPHT and MPCVD should exceed 2 inch, and wafers obtained by heterogeneous epitaxy and splicing methods should exceed 4 inch.
(b) Doping technology: There is a lack of effective n-type doping methods and a low concentration of p-type doping holes. The article has already mentioned the search for new growth directions to improve doping efficiency and the technology of achieving n-type doping through co-doping. In the future, it is expected to obtain p-type doping concentrations above 10^21^ cm^−3^ and n-type doping concentrations above 10^16^ cm^−3^ to achieve high-power applications.
(c) Reliability: The reliability and lifetime of diamond devices have not yet been fully proven. There is less research on reliability testing, which needs to be achieved by building more simulation models and testing actual devices.
(d) Thermal management and packaging: According to the research, diamond seems to be the only semiconductor with a sharp decrease in resistivity with temperature. While this is certainly an advantage, it also poses some problems in that the optimal operating state of the diamond device changes at different temperatures, which makes designing difficult. Due to this unique temperature characteristic, no encapsulation technology currently exists for diamonds. Electromagnetic compatibility (EMC) issues need to be considered. Special materials and designs are used to improve the reliability and long-term stability of the package and may include integrated thermal structures to help dissipate heat [243].
(e) Device performance: As summarized in this review, diamond devices need to further improve breakdown voltage and reduce R_on_. The current experimental device samples are small, and the parameters are not stable enough, and stable performance is required for commercial products. This will be achieved by refining doping techniques and introducing more power device structures, such as Insulated Gate Bipolar Transistors (IGBTs), resurf structures, and super-junction structures, which all rely on the realization of p-n junctions.
(f) Cost: This is a major obstacle to the commercialization of diamond. The current production cost of diamond is much higher than Si, SiC, and GaN, the mature semiconductor materials. The price of diamond materials used for semiconductor research is several thousand to tens of thousands of times higher than that of silicon materials.

**Figure 27:**
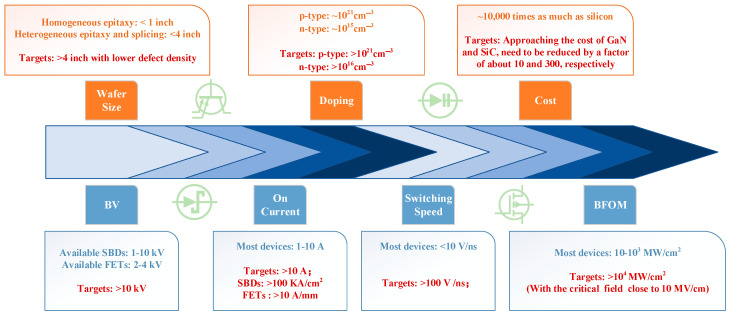
A parametric roadmap of diamond process and performance requirements, including the research status and expectations.

A simple roadmap for development is shown in Figure 27.

### 6.2. Commercialization Trends and Outlook

In terms of current trends, diamond is likely to be used in the future mainly in electric vehicle charging stations, solid state transformers [244], and other places where high temperatures, high power density, and high reliability are in demand, and as electric vehicles are currently being promoted around the world, the development of diamond is commercially promising. Data from Virtuemarket state that the global diamond semiconductor substrates market was valued at 151 million dollars in 2023 and is expected to reach a market size of 342 million dollars by the end of 2030. The market is expected to grow at a CAGR of 12.3% during the forecast period of 2024–2030 [245].

Some semiconductor companies have begun experimental diamond production. France’s Diamfab, which has four patents in the field of diamond epitaxy and doping, expects to achieve 4-inch wafer growth in 2025 [246]. The U.S.-based Akhan Semiconductor was founded in 2007 and pioneered the technology of low-temperature deposition of nanocrystalline diamond in 2013. This was followed by the establishment of the Miraj Diamond platform, the development of the growth of n-type diamond on Si, obtaining more than 1000 cm^2^/Vs mobility. In January 2024, the company’s founder Adam Khan announced the formation of a new company, Diamond Quanta, citing breakthroughs in diamond semiconductor fabrication and doping, which will be presenting the breakthroughs on August 20th and 21st at the upcoming International Materials Research Congress in Cancun, Mexico [247].

### 6.3. Conclusions

This article summarizes the development history of diamond materials and processes, summarizes the current development status of diamond devices, especially diodes and field-effect transistors, compares various diamond device parameters vertically, and compares them with devices prepared from other semiconductor materials (SiC, GaN, and β-Ga_2_O_3_) horizontally. It also analyzes the current advantages of diamond devices and the challenges faced in commercialization and their integration with existing technologies and proposes some solutions. In summary, to fully harness the potential of diamond, we need to address key challenges such as large-scale, high-quality, and single-crystal diamond material growth, diamond-compatible ion implantation techniques, and effective doping to realize both n- and p-type devices. In addition, integrating established power device design concepts like field plates, floating fields, and super-junctions into diamond technology has the potential to unlock even greater performance. By addressing these challenges through ongoing research and development, researchers can unlock the true potential of diamond and pave the way for its use in next-generation, high-performance power electronics, especially for high-temperature (>450 K), high-voltage (>10 kV), and high-BFOM (>10^4^ MW/cm^2^) applications.

## Figures and Tables

**Table 1:** Standard diamond terminations and electron affinity [93].

Terminal Type	H	O	F	Si	N
Symbol	C-H	C-O	C-F	C-Si	C-N
Electron affinity (eV)	−1.0	1.7	2.56	−0.86	3.46

**Table 2:** Contact potentials of different metals with diamonds.

Metal	Barrier Height (eV)	Contact Type
W [115] (annealing at 773 K)	0.45 ± 0.12	Schottky
Al [118] (at T = 294 K)	1.05	Schottky
Ni [103]	1.57	Schottky
Mo [119] (with O-Diamond)	1.61 ± 0.03~1.98 ± 0.02	Schottky
Cr [103]	0.73	Schottky
Zr [105] (with O-Diamond)	1.97	Schottky
Ti [112]	−0.63	Ohmic
Au (with H-Diamond) [116]	−0.19	Ohmic
Au (with O-Diamond) [116]	1.71	Schottky

**Table 3:** Diamond diode representative parameters.

Device	LSBDs	pVSBDs	VSBDs	SPND/SPIND
Current density	5.39 mA/mm at V = 40 V with T = 473 K [142]	1000 A/cm^2^ at V = 6 V with T = 300 K [104]	2980 A/cm^2^ at V = 8 V with T = 300 K [155]	>60 kA/cm^2^ at V = 6 V with T = 300 K [143]
Breakdown voltage	1651 V at T = 300 K [156]	1600 V at T = 300 K [76]	~10 kV under vacuum [121]	>50 V at T = 300 K [157]
Rectifying ratios	10^12^ at ±4 V with T = 300 K [158]	10^10^ at T = 300 K [130]	>10^10^ at ±10 V with T = 350 K [159] 10^11^ at ±10 V with T = 300 K [160]	>10^13^ at ±8 V with T = 300 K [161]

**Table 4:** Performance comparison of diamond field-effect tubes and bipolar transistors.

Device	Structural Characteristics	V_TH_ (V)	|I_D_MAX_| (mA/mm)	I_on_/I_off_	|BV|(V)	R_on_ (mΩ·cm^2^)	BFOM (MW/cm^2^)	Ref.
H-terminated MOSFETs	NO_2_ p-type doping	4.1	372	>10^7^	3659	13.48	173	[177]
	ALD-Al_2_O_3_ (10 nm @ 450 °C)	1.36	388	~2 × 10^7^	>50V	—	—	[229]
	NO_2_ p-type doping	3.9–4.1	680	~10^7^	2568	7.54	874.6	[39]
	V_2_O_5_ gate dielectric layer (10 nm)	1.7	375	~10^7^	—	16.8	—	[182]
	N-implanted region in channel	−2.5	5.4/2.7	>10^7^	531/1600	—	—	[228]
	ALD-Al_2_O_3_ (200 nm @ 450 °C with partial C-O channel)	−2.5~−4.0	5.2~18.2	10^8^	2021	—	—	[198]
Si-terminated MOSFETs	ALD-Al_2_O_3_ (100 nm @ 450 °C)	−5.6	311	>10^7^	150.2	—	—	[230]
Trench MOSFETs	Vertical U-trench	17.8	210	~10^6^	580	23	14.6	[231]
	Vertical V-trench	20.2~20.5	12,800	~10^7^	340	3.2	31.0	[221]
	Vertical U-trench with n-implanted/n-doped epitaxial layer	23.2/23.0	234/191	~10^8^	249/359	31/41	2/3.1	[220]
JFETs	Lateral p-n junctions	—	1.2	—	608	3.7	99.91	[223]
BJTs	On (001)	αT = 0.44, β = 0.79, diffusion length = 4.2 × 10^−5^ cm^3^ αT = 0.91, β = 10.1, diffusion length = 4.3 × 10^−5^ cm^3^						[212]
	On (111)							[232]
HBTs	p-n-p:lGaAs/GaAs/Diamond (e-b-c)	e-b:AlGaAs/GaAs n = 1.09, Ion/Ioff = 1.53 × 10^7^ at ±1.5 V				β ≈ 1		[213]
		b-c:GaAs/diamond n = 3.67, Ion/Ioff = 3.74 × 10^10^ at ±5.2V						

**Table 5:** Comparison of different wide bandgap and ultra-wide bandgap semiconductor devices with a focus on BFOM.

	Diodes				FETs/HEMTs			
	BV (kV)	Ron (mΩ·cm^2^)	BFOM (MW/cm^2^)	Ref.	BV (kV)	Ron (mΩ·cm^2^)	BFOM (MW/cm^2^)	Ref.
SiC	1.43	5.25	390	[235]	1.83	11	304.4	[236]
GaN	0.995	1.2	825	[237]	0.850	0.98	737	[238]
β-Ga_2_O_3_	8.32	5.24	1320	[239]	1.32	4.4	405	[240]
Diamond	0.7	5.89	332	[241]	2.57	7.54	874.6	[39]

## Data Availability

The data presented in this study are available in the article.

## References

[1] Baliga B.J. (1986). Modern Power Devices.

[2] Yue H. (2019). New progress in wide and ultra-wide bandgap semiconductor devices. Sci. Technol. Rev..

[3] Li C., Chen G., Li Y.Z., Zhou J.J., Li X. (2011). Application of B implantation in widegap semiconductor materials and devices. Res. Prog. SSE.

[4] Zhao L., Ge Q., Zhou Z., Yang B., Li Y. Research of high-power converter based on the wide band gap power semiconductor devices for rail transit electrical drive. Proceedings of the 2018 1st Workshop on Wide Bandgap Power Devices and Applications in Asia (WiPDA Asia).

[5] Zhang L., Yuan X., Wu X., Shi C., Zhang J., Zhang Y. (2019). Performance evaluation of high-power SiC MOSFET modules in comparison to Si IGBT modules. IEEE Trans. Power Electron..

[6] Dingle R., Störmer H.L., Gossard A.C., Wiegmann W. (1978). Electron mobilities in modulation-doped semiconductor heterojunction superlattices. Appl. Phys. Lett..

[7] Fujita S. (2015). Wide-bandgap semiconductor materials: For their full bloom. Jpn. J. Appl. Phys..

[8] Qin Y., Albano B., Spencer J., Lundh J.S., Wang B., Buttay C., Tadjer M., DiMarino C., Zhang Y. (2023). Thermal management and packaging of wide and ultra-wide bandgap power devices: A review and perspective. J. Phys. D Appl. Phys..

[9] Ramkumar M.S., Priya R., Rajakumari R.F., Valsalan P., Chakravarthi M.K., Latha G.C.P., Mathupriya S., Rajan K. (2022). Review and evaluation of power devices and semiconductor materials based on Si, SiC, and Ga-N. J. Nanomater..

[10] Adappa R., Suryanarayana K., Swathi Hatwar H., Ravikiran Rao M. Review of SiC based Power Semiconductor Devices and their Applications. Proceedings of the 2019 2nd International Conference on Intelligent Computing, Instrumentation and Control Technologies (ICICICT).

[11] O’Leary S.K., Foutz B.E., Shur M.S., Eastman L.F. (2006). Steady-state and transient electron transport within the III–V nitride semiconductors, GaN, AlN, and InN: A review. J. Mater. Sci. Mater. Electron..

[12] Kimoto T., Cooper J.A. (2014). Fundamentals of Silicon Carbide Technology: Growth, Characterization, Devices and Applications.

[13] Spaziani L., Lu L. Silicon, GaN and SiC: There’s room for all: An application space overview of device considerations. Proceedings of the 2018 IEEE 30th International Symposium on Power Semiconductor Devices and ICs (ISPSD).

[14] Soukiassian P., Amy F. (2005). Silicon carbide surface oxidation and SiO_2_/SiC interface formation investigated by soft X-ray synchrotron radiation. J. Electron. Spectros. Relat. Phenom..

[15] Buffolo M., Caria A., Piva F., Roccato N., Casu C., De Santi C., Trivellin N., Meneghesso G., Zanoni E., Meneghini M. (2022). Defects and reliability of GaN-based LEDs: Review and perspectives. Phys. Status Solidi (A).

[16] Zhang L.Q., Zhang S.M., Jiang D.S., Zhu J.J., Zhao D.G., Yang H. (2009). Characteristics of GaN based laser diode. Infrared Laser Eng..

[17] He J., Wei J., Yang S., Wang Y., Zhong K., Chen K.J. (2019). Frequency- and temperature-dependent gate reliability of Schottky-type p -GaN gate HEMTs. IEEE Trans. Electron. Devices.

[18] Rajashekara K., Akin B. A review of cryogenic power electronics—Status and applications. Proceedings of the 2013 International Electric Machines & Drives Conference.

[19] Higashiwaki M., Sasaki K., Kuramata A., Masui T., Yamakoshi S. (2012). Gallium oxide (Ga_2_O_3_) metal-semiconductor field-effect transistors on single-crystal β-Ga_2_O_3_ (010) substrates. Appl. Phys. Lett..

[20] Pearton S.J., Yang J., Cary P.H.I.V., Ren F., Kim J., Tadjer M.J., Mastro M.A. (2018). A review of Ga_2_O_3_ materials, processing, and devices. Appl. Phys. Rev..

[21] Umezawa H. (2018). Recent advances in diamond power semiconductor devices. Mater. Sci. Semicond. Process..

[22] Umezawa H., Nagase M., Kato Y., Shikata S.-I. (2012). High temperature application of diamond power device. Diam. Relat. Mater..

[23] Irokawa Y., Víllora E.A.G., Shimamura K. (2012). Shottky barrier diodes on AlN free-standing substrates. Jpn. J. Appl. Phys..

[24] Yuan Y., Hao W., Mu W., Wang Z., Chen X., Liu Q., Xu G., Wang C., Zhou H., Zou Y. (2021). Toward emerging gallium oxide semiconductors: A roadmap. Fundam. Res..

[25] Li L., Liao F., Hu X.T. (2020). The possibility of N-P codoping to realize P type β-Ga_2_O_3_. Superlattices Microstruct..

[26] Bell M.D., Leivo W.J. (1958). Rectification, photoconductivity, and photovoltaic effect in semiconducting Diamond. Phys. Rev..

[27] Liu M., Xu P.P., Zhang J.C., Liu B., Zhang L.W. (2020). A 4.2-to-5.4 GHz stacked GaAs HBT power amplifier for C-band applications. Circuit World.

[28] Perez G., Marechal A., Chicot G., Lefranc P., Jeannin P.O., Eon D., Rouger N. (2020). Diamond semiconductor performances in power electronics applications. Diam. Relat. Mater..

[29] Isberg J., Hammersberg J., Johansson E., Wikström T., Twitchen D.J., Whitehead A.J., Coe S.E., Scarsbrook G.A. (2002). High carrier mobility in single-crystal plasma-deposited diamond. Science.

[30] Yu X., Zhou J., Wang Y., Qiu F., Kong Y., Wang H., Chen T. (2019). Breakdown enhancement of diamond Schottky barrier diodes using boron implanted edge terminations. Diam. Relat. Mater..

[31] Liu E.K. (2017). Semiconductor Physics.

[32] Balmer R.S., Brandon J.R., Clewes S.L., Dhillon H.K., Dodson J.M., Friel I., Inglis P.N., Madgwick T.D., Markham M.L., Mollart T.P. (2009). Chemical vapour deposition synthetic diamond: Materials, technology and applications. J. Phys. Condens. Matter.

[33] Chu C.J., Hauge R.H., Margrave J.L., D’Evelyn M.P. (1992). Growth kinetics of (100), (110), and (111) homoepitaxial diamond films. Appl. Phys. Lett..

[34] Tsao J.Y., Chowdhury S., Hollis M.A., Jena D., Johnson N.M., Jones K.A., Kaplar R.J., Rajan S., Van de Walle C.G., Bellotti E. (2018). Ultrawide-bandgap semiconductors: Research opportunities and challenges. Adv. Electron. Mater..

[35] Bar-Cohen A., Albrecht J.D., Maurer J.J. Near-junction thermal management for wide bandgap devices. Proceedings of the 2011 IEEE Compound Semiconductor Integrated Circuit Symposium (CSICS).

[36] Chemical Semiconductors Akhan Semiconductor to Expand Market for Diamond Electronic Products [EB/OL]. https://www.compoundsemiconductorchina.net/labfab-news.asp?id=3608.

[37] Arnault J.-C., Saada S., Ralchenko V. (2022). Chemical vapor deposition single-crystal Diamond: A review. Phys. Status Solidi Rapid Res. Lett..

[38] Imanishi S., Horikawa K., Oi N., Okubo S., Kageura T., Hiraiwa A., Kawarada H. (2019). 3.8 W/mm RF power density for ALD Al_2_O_3_-based two-dimensional hole gas Diamond MOSFET operating at saturation velocity. IEEE Electron. Device Lett..

[39] Saha N.C., Kim S.-W., Oishi T., Kasu M. (2022). 875-MW/cm² low-resistance NO_2_ p-type doped chemical mechanical planarized Diamond MOSFETs. IEEE Electron. Device Lett..

[40] Sun J., Zhang Y., He D. (2000). A chemical adsorption growth model for hot filament chemical vapor deposition diamond. Diam. Relat. Mater..

[41] Zhang Y., Zang C., Ma H., Liang Z., Zhou L., Li S., Jia X. (2008). HPHT synthesis of large single crystal diamond doped with high nitrogen concentration. Diam. Relat. Mater..

[42] He Q., Su K., Zhang J., Ren Z., Xing Y., Zhang J., Lei Y., Hao Y. (2022). High mobility normally-OFF hydrogenated Diamond field effect transistors with BaF_2_ gate insulator formed by electron beam evaporator. IEEE Trans. Electron. Devices.

[43] Bundy F.P., Hall H.T., Strong H.M., Wentorfjun R.H. (1955). Man-made diamonds. Nature.

[44] Kamo M., Sato Y., Matsumoto S., Setaka N. (1983). Diamond synthesis from gas phase in microwave plasma. J. Cryst. Growth.

[45] Derjaguin B.V., Fedoseev D.V., Lukyanovich V.M., Spitzin B.V., Ryabov V.A., Lavrentyev A.V. (1968). Filamentary diamond crystals. J. Cryst. Growth.

[46] Sokol A.G., Pal’yanov Y.N. (2008). Diamond formation in the system MgO-SiO-HO-C at 7. 5 GPa and 1600 °C. Contrib. Mineral. Petrol..

[47] Fagan A.J., Luth R.W. (2011). Growth of diamond in hydrous silicate melts. Contrib. Mineral. Petrol..

[48] Shu G., Ralchenko V.G., Bolshakov A.P., Zavedeev E.V., Khomich A.A., Pivovarov P.A., Ashkinazi E.E., Konov V.I., Dai B., Han J. (2020). Evolution of surface relief of epitaxial diamond films upon growth resumption by microwave plasma chemical vapor deposition. CrystEngComm.

[49] May P.W. (2000). Diamond thin films: A 21st-century material. Philos. Trans. A Math. Phys. Eng. Sci..

[50] Vikharev A.L., Lobaev M.A., Gorbachev A.M., Radishev D.B., Isaev V.A., Bogdanov S.A. (2020). Investigation of homoepitaxial growth by microwave plasma CVD providing high growth rate and high quality of diamond simultaneously. Mater. Today Commun..

[51] Widmann C.J., Müller-Sebert W., Lang N., Nebel C.E. (2016). Homoepitaxial growth of single crystalline CVD-diamond. Diam. Relat. Mater..

[52] Mallik A.K., Bysakh S., Pal K.S., Dandapat N., Guha B.K., Datta S., Basu D. (2013). Large area deposition of polycrystalline Diamond coatings by microwave plasma CVD. Trans. Indian Ceram. Soc..

[53] Dai Z., Bednarski-Meinke C., Loloee R., Golding B. (2003). Epitaxial (100) iridium on A-plane sapphire: A system for wafer-scale diamond heteroepitaxy. Appl. Phys. Lett..

[54] Nad S., Charris A., Asmussen J. (2016). MPACVD growth of single crystalline diamond substrates with PCD rimless and expanding surfaces. Appl. Phys. Lett..

[55] Yamada H., Chayahara A., Mokuno Y., Kato Y., Shikata S. (2014). A 2-in. mosaic wafer made of a single-crystal diamond. Appl. Phys. Lett..

[56] Liang Q., Chin C.Y., Lai J., Yan C.S., Meng Y., Mao H.K., Hemley R.J. (2009). Enhanced growth of high quality single crystal diamond by microwave plasma assisted chemical vapor deposition at high gas pressures. Appl. Phys. Lett..

[57] Su Y., Li H.D., Cheng S.H., Zhang Q., Wang Q.L., Lv X.Y., Zou G.T., Pei X.Q., Xie J.G. (2012). Effect of NO on high-rate homoepitaxial growth of CVD single crystal diamonds. J. Cryst. Growth.

[58] Zhang Q., Li H.D., Cheng S.H., Wang Q.L., Li L.A., Lv X.Y., Zou G.T. (2011). The effect of CO_2_ on the high-rate homoepitaxial growth of CVD single crystal diamonds. Diam. Relat. Mater..

[59] Schreck M., Gsell S., Brescia R., Fischer M. (2017). Ion bombardment induced buried lateral growth: The key mechanism for the synthesis of single crystal diamond wafers. Sci. Rep..

[60] Toros A., Kiss M., Graziosi T., Mi S., Berrazouane R., Naamoun M., Vukajlovic Plestina J., Gallo P., Quack N. (2020). Reactive ion etching of single crystal diamond by inductively coupled plasma: State of the art and catalog of recipes. Diam. Relat. Mater..

[61] Wang J.S., Wan L., Chen J., Yan J.W. (2016). Surface patterning of synthetic diamond crystallites using nickel powder. Diam. Relat. Mater..

[62] Hiromu Shiomi H.S. (1997). Reactive ion etching of Diamond in O_2_ and CF_4_ plasma, and fabrication of porous Diamond for field emitter cathodes. Jpn. J. Appl. Phys..

[63] Whetten T.J., Armstead A.A., Grzybowski T.A., Ruoff A.L. (1984). Etching of diamond with argon and oxygen ion beams. J. Vac. Sci. Technol. A.

[64] Grogan D.F., Zhao T., Bovard B.G., Macleod H.A. (1992). Planarizing technique for ion-beam polishing of diamond films. Appl. Opt..

[65] Lee C.L., Gu E., Dawson M.D., Friel I., Scarsbrook G.A. (2008). Etching and micro-optics fabrication in diamond using chlorine-based inductively-coupled plasma. Diam. Relat. Mater..

[66] Yamada T., Yoshikawa H., Uetsuka H., Kumaragurubaran S., Tokuda N., Shikata S.-I. (2007). Cycle of two-step etching process using ICP for diamond MEMS applications. Diam. Relat. Mater..

[67] Challier M., Sonusen S., Barfuss A., Rohner D., Riedel D., Koelbl J., Ganzhorn M., Appel P., Maletinsky P., Neu E. (2018). Advanced fabrication of single-crystal Diamond membranes for quantum technologies. Micromachines.

[68] Forsberg P., Karlsson M. (2013). High aspect ratio optical gratings in diamond. Diam. Relat. Mater..

[69] Wang J., Enlund J., Isberg J., Karlsson M., Nikolajeff F., Olsson J., Twitchen D.J. (2005). Anisotropic dry etching of boron doped single crystal CVD diamond. Carbon.

[70] Khanaliloo B., Mitchell M., Hryciw A.C., Barclay P.E. (2015). High-Q/V monolithic Diamond microdisks fabricated with quasi-isotropic etching. Nano Lett..

[71] Mitchell M., Lake D.P., Barclay P.E. (2019). Realizing Q> 300 000 in diamond microdisks for optomechanics via etch optimization. APL Photonics.

[72] Leech P.W., Reeves G.K., Holland A. (2001). Reactive ion etching of diamond in CF_4_, O_2_, O_2_ and Ar-based mixtures. J. Mater. Sci..

[73] Ando Y., Nishibayashi Y., Kobashi K., Hirao T., Oura K. (2002). Smooth and high-rate reactive ion etching of diamond. Diam. Relat. Mater..

[74] Field J.E. (2012). The mechanical and strength properties of diamond. Rep. Prog. Phys..

[75] Monflier R., Isoird K., Cazarre A., Tasselli J., Servel A., Achard J., Eon D., Birnbaum M.J.V. (2017). Diamond Schottky diodes operating at 473 K. EPE J..

[76] Kumaresan R., Umezawa H., Tatsumi N., Ikeda K., Shikata S. (2009). Device processing, fabrication and analysis of diamond pseudo-vertical Schottky barrier diodes with low leak current and high blocking voltage. Diam. Relat. Mater..

[77] Wang J., Shao G., Li Q., Chen G., Yan X., Song Z., Wang Y., Wang R., Wang W., Fan S. (2022). Vertical Diamond trench MOS barrier Schottky diodes with high breakdown voltage. IEEE Trans. Electron. Devices.

[78] Fu Y., Bi T., Chang Y.H., Xu R.M., Xu Y.H., Kawarada H. (2023). Oxidized-Silicon-Terminated Diamond p-FETs With SiO_2-_Filling Shallow Trench Isolation Structures. IEEE Electron. Device Lett..

[79] Crawford K.G., Maini I., Macdonald D.A., Moran D.A.J. (2021). Surface transfer doping of diamond: A review. Prog. Surf. Sci..

[80] Masante C., Pernot J., Letellier J., Eon D., Rouger N. 175V, >5.4 MV/cm, 50 mΩ·cm^2^ at 250 °C Diamond MOSFET and its reverse conduction. Proceedings of the 2019 31st International Symposium on Power Semiconductor Devices and ICs (ISPSD).

[81] Lagrange J.P., Deneuville A., Gheeraert E. (1998). Activation energy in low compensated homoepitaxial boron-doped diamond films. Diam. Relat. Mater..

[82] Pernot J., Volpe P.N., Omnès F., Muret P., Mortet V., Haenen K., Teraji T. (2010). Hall hole mobility in boron-doped homoepitaxial diamond. Phys. Rev. B Condens. Matter Mater. Phys..

[83] Barjon J., Chikoidze E., Jomard F., Dumont Y., Pinault-Thaury M.A., Issaoui R., Brinza O., Achard J., Silva F. (2012). Homoepitaxial boron-doped diamond with very low compensation: Homoepitaxial boron-doped diamond. Phys. Status Solidi (A).

[84] Kitagoh S., Okada R., Kawano A., Watanabe M., Takano Y., Yamaguchi T., Chikyow T., Kawarada H. (2010). Cross-sectional TEM study and film thickness dependence of Tc in heavily boron-doped superconducting diamond. Physica C.

[85] Tsubouchi N., Ogura M., Chayahara A., Okushi H. (2008). Formation of a heavily B doped diamond layer using an ion implantation technique. Diam. Relat. Mater..

[86] Tshepe T., Prins J.F., Hoch M.J.R. (1999). Metal–insulator transition in boron-ion implanted type IIa diamond. Diam. Relat. Mater..

[87] Childress L., Hanson R. (2013). Diamond NV centers for quantum computing and quantum networks. MRS Bull..

[88] Katamune Y., Mori D., Arikawa D., Izumi A., Shimaoka T., Ichikawa K., Koizumi S. (2020). n-Type doping of diamond by hot-filament chemical vapor deposition growth with phosphorus incorporation. Appl. Phys. A-Mater..

[89] Jones R., Lowther J.E., Goss J. (1996). Limitations to n-type doping in diamond: The phosphorus-vacancy complex. Appl. Phys. Lett..

[90] Prawer S., Uzan-Saguy C., Braunstein G., Kalish R. (1993). Can n-type doping of diamond be achieved by Li or Na ion implantation?. Appl. Phys. Lett..

[91] Zeisel R., Nebel C.E., Stutzmann M., Sternschulte H., Schreck M., Stritzker B. (2000). Photoconductivity study of Li doped homoepitaxially grown CVD Diamond. Physica Status Solidi A Appl. Res..

[92] Su H., Wang J.H., Xiong L.W., Liu P.F., Jiang C. (2011). Semiconductor application of doped nano-crystalline diamond film. J. Wuhan Inst. Technol..

[93] Kawai S., Yamano H., Sonoda T., Kato K., Buendia J.J., Kageura T., Fukuda R., Okada T., Tanii T., Higuchi T. (2019). Nitrogen-terminated Diamond surface for nanoscale NMR by shallow nitrogen-vacancy centers. J. Phys. Chem. C Nanomater. Interfaces.

[94] Strobel P., Riedel M., Ristein J., Ley L. (2004). Surface transfer doping of diamond. Nature.

[95] Fu Y. (2022). Study on Hydrogen-Terminated and Silicon-Terminated Diamond MOSFET. Ph.D. Thesis.

[96] Lv X., Wang W., Wang Y., Chen G., He S., Zhang M., Wang H. (2023). Hydrogen-terminated single crystal Diamond MOSFET with a bilayer dielectric of Gd_2_O_3_/Al_2_O_3_. Crystals.

[97] Hu W.X., Yu X.X., Tao T., Chen K., Ye Y.C., Zhou J.J., Xie Z.L., Yan Y., Liu B., Zhang R. (2023). H-Terminated Diamond MOSFETs on High-Quality Diamond Film Grown by MPCVD. Crystals.

[98] Yagi I., Notsu H., Kondo T., Tryk D.A., Fujishima A. (1999). Electrochemical selectivity for redox systems at oxygen-terminated diamond electrodes. J. Electroanal. Chem..

[99] Schenk A., Tadich A., Sear M., O’Donnell K.M., Ley L., Stacey A., Pakes C. (2015). Formation of a silicon terminated (100) diamond surface. Appl. Phys. Lett..

[100] Fu Y., Chang Y.H., Kono S., Hiraiwa A., Kanehisa K., Zhu X.H., Xu R.M., Xu Y.H., Kawarada H. (2022). −10 V Threshold Voltage High-Performance Normally-C-Si Diamond MOSFET Formed by p-Diamond-First and Silicon Molecular Beam Deposition Approaches. IEEE Trans. Electron. Devices.

[101] Qiao P., Liu K., Zhang S., Su Z., Dai B., Han J., Zhu J. (2022). Origin of two-dimensional hole gas formation on Si-treated diamond surfaces: Surface energy band diagram perspective. Appl. Surf. Sci..

[102] Peng B., Li Q., Zhang S.M., Fan S.W., Wang R.Z., Wang H.X. (2023). Research Progress of Diamond Schottky Barrier Diodes. J. Synth. Cryst..

[103] Koné S., Schneider H., Isoird K., Thion F., Achard J., Issaoui R., Msolli S., Alexis J. (2012). An assessment of contact metallization for high power and high temperature diamond Schottky devices. Diam. Relat. Mater..

[104] Mönch W. (1994). Barrier heights of metal contacts on H-terminated Diamond: Explanation by metal-induced gap states and interface dipoles. EPL.

[105] Piñero J.C., Araújo D., Fiori A., Traoré A., Villar M.P., Eon D., Muret P., Pernot J., Teraji T. (2017). Atomic composition of WC/ and Zr/O-terminated diamond Schottky interfaces close to ideality. Appl. Surf. Sci..

[106] Traoré A., Muret P., Fiori A., Eon D., Gheeraert E., Pernot J. (2014). Zr/oxidized diamond interface for high power Schottky diodes. Appl. Phys. Lett..

[107] Zhao D., Liu Z., Wang J., Liang Y., Nauman M., Fu J., Wang Y.-F., Fan S., Wang W., Wang H.-X. (2018). Fabrication of dual-termination Schottky barrier diode by using oxygen-/fluorine-terminated diamond. Appl. Surf. Sci..

[108] Zhao D., Liu Z., Wang J., Yi W., Wang R., Wang K., Wang H. (2019). Performance improved vertical Diamond Schottky barrier diode with fluorination-termination structure. IEEE Electron. Device Lett..

[109] Peng B., Wang Y.Y., Liu X.Q., Zhen C.M., Gong H.X., Yan Z.J., Yang Y.H., Ma S.Y. (2000). Ohmic contacts and interface properties of Au/Ti/p-diamnond prepared by r.f. sputtering. Surf. Interface Anal..

[110] Hoff H.A., Waytena G.L., Vold C.L., Suehle J.S., Isaacson I.P., Rebbert M.L., Ma D.I., Harris K. (1996). Ohmic contacts to semiconducting diamond using a Ti/Pt/Au trilayer metallization scheme. Diam. Relat. Mater..

[111] Kono S., Teraji T., Kodama H., Ichikawa K., Ohnishi S., Sawabe A. (2015). Direct determination of the barrier height of Ti-based ohmic contact on p-type diamond (001). Diam. Relat. Mater..

[112] Li Q., Wang J., Shao G.Q., Chen G.Q., He S., Zhang Q.W., Zhang S.M., Wang R.Z., Fan S.W., Wang H.X. (2022). Breakdown Voltage Enhancement of Vertical Diamond Schottky Barrier Diode with Annealing Method and Al_2_O_3_ Field Plate Structure. IEEE Electron. Device Lett..

[113] Shao G., Wang J., Liu Z., Wang Y., Wang W., Wang H.-X. (2021). Performance-improved vertical Zr/Diamond Schottky barrier diode with lanthanum hexaboride interfacial layer. IEEE Electron. Device Lett..

[114] Muret P., Volpe P.N., Tran-Thi T.N., Pernot J., Hoarau C., Omnès F., Teraji T. (2011). Schottky diode architectures on p-type diamond for fast switching, high forward current density and high breakdown field rectifiers. Diam. Relat. Mater..

[115] Zhao D., Li F.N., Liu Z.C., Chen X.D., Wang Y.F., Shao G.Q., Zhu T.F., Zhang M.H., Zhang J.W., Wang J.J. (2018). Effects of rapid thermal annealing on the contact of tungsten/p-diamond. Appl. Surf. Sci..

[116] Li F., Zhang J., Wang X., Zhang M., Wang H. (2017). Barrier heights of Au on Diamond with different terminations determined by X-ray photoelectron spectroscopy. Coatings.

[117] Kono S., Teraji T., Takeuchi D., Ogura M., Kodama H., Sawabe A. (2017). Direct determination of the barrier height of Au ohmic-contact on a hydrogen-terminated diamond (001) surface. Diam. Relat. Mater..

[118] Evans D.A., Roberts O.R., Vearey-Roberts A.R., Langstaff D.P., Twitchen D.J., Schwitters M. (2007). Direct observation of Schottky to Ohmic transition in Al-diamond contacts using real-time photoelectron spectroscopy. Appl. Phys. Lett..

[119] Woo K., Malakoutian M., Saraswat D., Bian Z., Hardy A., Muehle M., Grotjohn T.A., Chowdhury S. (2024). Control of Schottky barrier height in diamond using UV-generated ozone and its effect on barrier inhomogeneity and temperature dependent properties. Diam. Relat. Mater..

[120] Volpe P.-N., Muret P., Pernot J., Omnès F., Teraji T., Jomard F., Planson D., Brosselard P., Dheilly N., Vergne B. (2010). High breakdown voltage Schottky diodes synthesized on p-type CVD diamond layer. Phys. Status Solidi (A).

[121] Volpe P.-N., Muret P., Pernot J., Omnès F., Teraji T., Koide Y., Jomard F., Planson D., Brosselard P., Dheilly N. (2010). Extreme dielectric strength in boron doped homoepitaxial diamond. Appl. Phys. Lett..

[122] Bormashov V.S., Terentiev S.A., Buga S.G., Tarelkin S.A., Volkov A.P., Teteruk D.V., Kornilov N.V., Kuznetsov M.S., Blank V.D. (2017). Thin large area vertical Schottky barrier diamond diodes with low on-resistance made by ion-beam assisted lift-off technique. Diam. Relat. Mater..

[123] Zhao D., Hu C., Liu Z., Wang H.-X., Wang W., Zhang J. (2017). Diamond MIP structure Schottky diode with different drift layer thickness. Diam. Relat. Mater..

[124] Wang J., Zhao D., Shao G., Liu Z., Chang X., Chen G., Wang W., Yi W., Wang K., Wang H. (2021). Fabrication of dual-barrier planar structure Diamond Schottky diodes by rapid thermal annealing. IEEE Trans. Electron. Devices.

[125] Baliga B.J. (1984). The pinch rectifier: A low-forward-drop high-speed power diode. IEEE Electron. Device Lett..

[126] Umezawa H., Nagase M., Kato Y., Shikata S. (2012). Diamond vertical Schottky barrier diode with Al_2_O_3_ field plate. Mater. Sci. For..

[127] Driche K., Rugen S., Kaminski N., Umezawa H., Okumura H., Gheeraert E. (2018). Electric field distribution using floating metal guard rings edge-termination for Schottky diodes. Diam. Relat. Mater..

[128] Wang J., Zhao D., Wang W., Zhang X., Wang Y., Chang X., Liu Z., Fu J., Wang K., Wang H.-X. (2019). Diamond Schottky barrier diodes with floating metal rings for high breakdown voltage. Mater. Sci. Semicond. Process..

[129] Koizumi S., Umezawa H., Pernot J., Suzuki M. (2018). Power Electronics Device Applications of Diamond Semiconductors.

[130] Hazdra P., Laposa A., Soban Z., Taylor A., Lambert N., Povolny V., Kroutil J., Gedeonov Z., Hubik P., Mortet V. (2022). Pseudo-vertical Mo/Au Schottky diodes on {113} oriented boron doped homoepitaxial diamond layers. Diam. Relat. Mater..

[131] Hanada T., Ohmagari S., Kaneko J.H., Umezawa H. (2020). High yield uniformity in pseudo-vertical diamond Schottky barrier diodes fabricated on half-inch single-crystal wafers. Appl. Phys. Lett..

[132] Mortet V., Taylor A., Lambert N., Gedeonová Z., Fekete L., Lorinčik J., Klimša L., Kopeček J., Hubík P., Šobáň Z. (2021). Properties of boron-doped (113) oriented homoepitaxial diamond layers. Diam. Relat. Mater..

[133] Mehrotra M., Baliga B.J. (1995). Trench MOS Barrier Schottky (TMBS) rectifier: A Schottky rectifier with higher than parallel plane breakdown voltage. Solid State Electron..

[134] Zhu L., Chow T.P., Jones K.A., Agarwal A. (2006). Design, fabrication, and characterization of low forward drop, low leakage, 1-kV 4H-SiC JBS rectifiers. IEEE Trans. Electron. Devices.

[135] Khemka V., Ananthan V., Chow T.P. (2000). A fully planarized 4H-SiC trench MOS barrier Schottky (TMBS) rectifier. IEEE Electron. Device Lett..

[136] Hasegawa K., Nishio G., Yasunishi K., Tanaka N., Murakami N., Oka T. (2017). Vertical GaN trench MOS barrier Schottky rectifier maintaining low leakage current at 200 °C with blocking voltage of 750 V. Appl. Phys. Express.

[137] Sasaki K., Wakimoto D., Thieu Q.T., Koishikawa Y., Kuramata A., Higashiwaki M., Yamakoshi S. Demonstration of Ga_2_O_3_ Trench MOS-Type Schottky Barrier Diodes. Proceedings of the 2017 75th Annual Device Research Conference (DRC).

[138] Li W., Nomoto K., Hu Z., Tanen N., Sasaki K., Kuramata A., Jena D., Xing H.G. 1.5 kV Vertical Ga_2_O_3_ Trench-MIS Schottky Barrier Diodes. Proceedings of the 2018 76th Device Research Conference (DRC).

[139] Lin W., Wang Q., Lv X., Li L., Zou G. (2023). Design of trench Schottky barrier diode on diamond for obtaining high performance. Diam. Relat. Mater..

[140] Teraji T., Koizumi S., Koide Y., Ito T. (2007). Electric field breakdown of lateral Schottky diodes of Diamond. Jpn. J. Appl. Phys..

[141] Koizumi S., Umezawa H., Pernot J., Suzuki M. (2018). Key technologies for device fabrications and materials characterizations. Power Electronics Device Applications of Diamond Semiconductors.

[142] Han Z., Bayram C. (2023). Diamond p-type lateral Schottky barrier diodes with high breakdown voltage (4612 V at 0.01 mA/mm). IEEE Electron. Device Lett..

[143] Makino T., Kato H., Tokuda N., Ogura M., Takeuchi D., Oyama K., Tanimoto S., Okushi H., Yamasaki S. (2010). Diamond Schottky-pn diode without trade-off relationship between on-resistance and blocking voltage. Phys. Status Solidi (A).

[144] Kubovic M., El-Hajj H., Butler J.E., Kohn E. (2007). Diamond merged diode. Diam. Relat. Mater..

[145] Hitchcock C., Chow T.P. (2020). Degradation of forward current density with increasing blocking voltage in diamond Schottky-pn diodes. Diam. Relat. Mater..

[146] Suzuki M., Sakai T., Makino T., Kato H., Takeuchi D., Ogura M., Okushi H., Yamasaki S. (2013). Electrical characterization of diamond PiN diodes for high voltage applications: Electrical characterization of diamond PiN diodes. Phys. Status Solidi (A).

[147] Saremi M., Hathwar R., Dutta M., Koeck F.A.M., Nemanich R.J., Chowdhury S., Goodnick S.M. (2017). Analysis of the reverse I-V characteristics of diamond-based PIN diodes. Appl. Phys. Lett..

[148] Makino T., Tanimoto S., Kato H., Tokuda N., Ogura M., Takeuchi D., Oyama K., Ohashi H., Okushi H., Yamasaki S. (2009). Diamond Schottky p-n diode with high forward current density. Phys. Status Solidi (A).

[149] Makino T., Oyama K., Kato H., Takeuchi D., Ogura M., Okushi H., Yamasaki S. (2014). Diamond electronic devices fabricated using heavily doped hopping p^+^ and n^+^ layers. Jpn. J. Appl. Phys..

[150] Kato H., Yamasaki S., Okushi H. (2005). N -type doping of (001)-oriented single-crystalline diamond by phosphorus. Appl. Phys. Lett..

[151] Koizumi S., Kamo M., Sato Y., Ozaki H., Inuzuka T. (1997). Growth and characterization of phosphorous doped 111 homoepitaxial diamond thin films. Appl. Phys. Lett..

[152] Donato N., Rouger N., Pernot J., Longobardi G., Udrea F. (2020). Diamond power devices: State of the art, modelling, figures of merit and future perspective. J. Phys. D Appl. Phys..

[153] Matsumae T., Kurashima Y., Umezawa H., Tanaka K., Ito T., Watanabe H., Takagi H. (2020). Low-temperature direct bonding of β-Ga_2_O_3_ and diamond substrates under atmospheric conditions. Appl. Phys. Lett..

[154] Sittimart P., Ohmagari S., Matsumae T., Umezawa H., Yoshitake T. (2021). Diamond/β-Ga_2_O_3_ pn heterojunction diodes fabricated by low-temperature direct-bonding. AIP Adv..

[155] Kumaresan R., Umezawa H., Shikata S. (2010). Vertical structure Schottky barrier diode fabrication using insulating diamond substrate. Diam. Relat. Mater..

[156] Saha N.C., Irie Y., Seki Y., Hoshino Y., Nakata J., Kim S.-W., Oishi T., Kasu M. (2023). 1651-V all-ion-implanted Schottky barrier diode on heteroepitaxial Diamond with 3.6 × 10⁵ on/off ratio. IEEE Electron Device Lett..

[157] Dutta M., Koeck F.A.M., Hathwar R., Goodnick S.M., Nemanich R.J., Chowdhury S. (2016). Demonstration of Diamond-based Schottky p-i-n diode with blocking voltage >500 V. IEEE Electron. Device Lett..

[158] Kawashima H., Noguchi H., Matsumoto T., Kato H., Ogura M., Makino T., Shirai S., Takeuchi D., Yamasaki S. (2015). Electronic properties of diamond Schottky barrier diodes fabricated on silicon-based heteroepitaxially grown diamond substrates. Appl. Phys. Express.

[159] Shao G., Wang J., Zhang S., Wang Y., Wang W., Wang H.-X. (2023). Effect of rapid thermal annealing on performances of vertical boron-doped diamond Schottky diode with LaB6 interlayer. Diam. Relat. Mater..

[160] Li Q., Wang J., Chen G., He S., Zhang Q., Zhang S., Wang R., Fan S., Wang H.-X. (2023). Breakdown voltage enhancement of vertical diamond Schottky barrier diodes by selective growth nitrogen-doped diamond field plate. Diam. Relat. Mater..

[161] Matsumoto T., Mukose T., Makino T., Takeuchi D., Yamasaki S., Inokuma T., Tokuda N. (2017). Diamond Schottky-pn diode using lightly nitrogen-doped layer. Diam. Relat. Mater..

[162] Chakrapani V., Angus J.C., Anderson A.B., Wolter S.D., Stoner B.R., Sumanasekera G.U. (2007). Charge transfer equilibria between diamond and an aqueous oxygen electrochemical redox couple. Science.

[163] Sato H., Kasu M. (2012). Electronic properties of H-terminated diamond during NO_2_ and O adsorption and desorption. Diam. Relat. Mater..

[164] Gi R., Kazuhiro Tashiro K.T., Seiichi Tanaka S.T., Takao Fujisawa T.F., Hideki Kimura H.K., Tateki Kurosu T.K., Masamori Iida M.I. (1999). Hall effect measurements of surface conductive layer on undoped Diamond films in NO_2_ and NH_3_ atmospheres. Jpn. J. Appl. Phys..

[165] Sque S.J., Jones R., Öberg S., Briddon P.R. (2006). Transfer doping of diamond: The use of and to effect p-type surface conductivity. Phys. B Condens. Matter.

[166] Russell S.A.O., Cao L., Qi D., Tallaire A., Crawford K.G., Wee A.T.S., Moran D.A.J. (2013). Surface transfer doping of diamond by MoO_3_: A combined spectroscopic and Hall measurement study. Appl. Phys. Lett..

[167] Crawford K.G., Cao L., Qi D., Tallaire A., Limiti E., Verona C., Wee A.T.S., Moran D.A.J. (2016). Enhanced surface transfer doping of diamond by V_2_O_5_ with improved thermal stability. Appl. Phys. Lett..

[168] Kawarada H., Aoki M., Ito M. (1994). Enhancement mode metal-semiconductor field effect transistors using homoepitaxial diamonds. Appl. Phys. Lett..

[169] Gluche P., Aleksov A., Vescan A., Ebert W., Kohn E. (1997). Diamond surface-channel FET structure with 200 V breakdown voltage. IEEE Electron. Device Lett..

[170] Kawarada H., Wild C., Herres N., Koidl P., Mizuochi Y., Hokazono A., Nagasawa H. (1998). Surface morphology and surface p-channel field effect transistor on the heteroepitaxial diamond deposited on inclined β-SiC(001) surfaces. Appl. Phys. Lett..

[171] Hokazono A., Tsugawa K., Umezana H., Kitatani K., Kawarada H. (1999). Surface p-channel metal-oxide-semiconductor field effect transistors fabricated on hydrogen terminated (001) surfaces of diamond. Solid State Electron..

[172] Kasu M. (2004). Influence of epitaxy on the surface conduction of diamond film. Diam. Relat. Mater..

[173] Hirama K., Takayanagi H., Yamauchi S., Jingu Y., Umezawa H., Kawarada H. High-performance p-channel diamond MOSFETs with alumina gate insulator. Proceedings of the 2007 IEEE International Electron Devices Meeting.

[174] Hiraiwa A., Daicho A., Kurihara S., Yokoyama Y., Kawarada H. (2012). Refractory two-dimensional hole gas on hydrogenated diamond surface. J. Appl. Phys..

[175] Kawarada H., Yamada T., Xu D., Tsuboi H., Saito T., Hiraiwa A. Wide temperature (10 K–700 K) and high voltage (~1000 V) operation of C-H diamond MOSFETs for power electronics application. Proceedings of the 2014 IEEE International Electron Devices Meeting.

[176] Kawarada H., Yamada T., Xu D., Tsuboi H., Kitabayashi Y., Matsumura D., Shibata M., Kudo T., Inaba M., Hiraiwa A. (2017). Durability-enhanced two-dimensional hole gas of C-H diamond surface for complementary power inverter applications. Sci. Rep..

[177] Saha N.C., Kim S.-W., Koyama K., Oishi T., Kasu M. (2023). 3659-V NO_2_ p-Type Doped Diamond MOSFETs on Misoriented Heteroepitaxial Diamond Substrates. IEEE Electron Device Lett..

[178] Kudara K., Imanishi S., Hiraiwa A., Komatsuzaki Y., Yamaguchi Y., Kawamura Y., Shinjo S., Kawarada H. (2021). High output power density of 2DHG Diamond MOSFETs with thick ALD-Al_2_O_3_. IEEE Trans. Electron. Devices.

[179] Wade T., Geis M.W., Fedynyshyn T.H., Vitale S.A., Varghese J.O., Lennon D.M., Grotjohn T.A., Nemanich R.J., Hollis M.A. (2017). Effect of surface roughness and H-termination chemistry on diamond’s semiconducting surface conductance. Diam. Relat. Mater..

[180] Kubovic M., Kasu M., Kageshima H., Maeda F. (2010). Electronic and surface properties of H-terminated diamond surface affected by NO_2_ gas. Diam. Relat. Mater..

[181] Liu J., Ohsato H., Liao M., Imura M., Watanabe E., Koide Y. (2017). Logic circuits with hydrogenated Diamond field-effect transistors. IEEE Electron. Device Lett..

[182] Macdonald D.A., Crawford K.G., Tallaire A., Issaoui R., Moran D.A.J. (2018). Performance enhancement of Al_2_O_3_/H-Diamond MOSFETs utilizing vacuum annealing and V_2_O_5_ as a surface electron acceptor. IEEE Electron. Device Lett..

[183] Wang W., Wang Y., Zhang M., Wang R., Chen G., Chang X., Lin F., Wen F., Jia K., Wang H.-X. (2020). An enhancement-mode hydrogen-terminated Diamond field-effect transistor with lanthanum hexaboride gate material. IEEE Electron. Device Lett..

[184] Liu J.W., Liao M.Y., Imura M., Matsumoto T., Shibata N., Ikuhara Y., Koide Y. (2015). Control of normally on/off characteristics in hydrogenated diamond metal-insulator-semiconductor field-effect transistors. J. Appl. Phys..

[185] Liu J.W., Oosato H., Liao M.Y., Koide Y. (2017). Enhancement-mode hydrogenated diamond metal-oxide-semiconductor field-effect transistors with Y_2_O_3_ oxide insulator grown by electron beam evaporator. Appl. Phys. Lett..

[186] Ren Z., Zhang J., Zhang J., Zhang C., Xu S., Li Y., Hao Y. (2017). Diamond field effect transistors with MoO_3_Gate dielectric. IEEE Electron. Device Lett..

[187] Ren Z., Zhang J., Zhang J., Zhang C., Chen D., Yang P., Li Y., Hao Y. (2017). Polycrystalline Diamond MOSFET with MoO_3_ Gate Dielectric and Passivation Layer. IEEE Electron. Device Lett..

[188] Tordjman M., Weinfeld K., Kalish R. (2017). Boosting surface charge-transfer doping efficiency and robustness of diamond with WO_3_ and ReO_3_. Appl. Phys. Lett..

[189] Liu J.W., Liao M.Y., Imura M., Koide Y. (2013). Normally-off HfO_2_-gated diamond field effect transistors. Appl. Phys. Lett..

[190] Ren Z., Lv D., Xu J., Su K., Zhang J., Wang D., Wu Y., Zhang J., Hao Y. (2020). Performance of H-diamond MOSFETs with high temperature ALD grown HfO_2_ dielectric. Diam. Relat. Mater..

[191] Chen Z., Yu X., Mao S., Zhou J., Kong Y., Chen T., Xu R., Yan B., Xu Y. (2024). Hydrogen-terminated Diamond field-effect transistors with 1011 ON/ OFF ratio using an Al_2_O_3_/HfO_2_ stacked passivation layer. IEEE Trans. Electron Devices.

[192] Wang F., Chen G., Shao G., Wang W., Zhang M., Wang Y., Zhang Q., He S., Hu W., Wang H. (2024). Electrical Characteristics of H-Diamond Transistors With ZrO_2_/Zr Stacked Dielectrics Deposited by Electron Beam Evaporation. IEEE Trans. Electron. Devices.

[193] Zhao J., Liu J., Sang L., Liao M., Coathup D., Imura M., Shi B., Gu C., Koide Y., Ye H. (2016). Assembly of a high-dielectric constant thin TiO_x_ layer directly on H-terminated semiconductor diamond. Appl. Phys. Lett..

[194] Daligou G., Pernot J. (2020). 2D hole gas mobility at diamond/insulator interface. Appl. Phys. Lett..

[195] Sasama Y., Kageura T., Imura M., Watanabe K., Taniguchi T., Uchihashi T., Takahide Y. (2021). High-mobility p-channel wide-bandgap transistors based on hydrogen-terminated diamond/hexagonal boron nitride heterostructures. Nat. Electron..

[196] Mirabedini P.S., Debnath B., Neupane M.R., Alex Greaney P., Glen Birdwell A., Ruzmetov D., Crawford K.G., Shah P., Weil J., Ivanov T.G. (2020). Structural and electronic properties of 2D (graphene, hBN)/H-terminated diamond (100) heterostructures. Appl. Phys. Lett..

[197] Liu J.W., Liao M.Y., Imura M., Banal R.G., Koide Y. (2017). Deposition of TiO_2_/Al_2_O_3_ bilayer on hydrogenated diamond for electronic devices: Capacitors, field-effect transistors, and logic inverters. J. Appl. Phys..

[198] Kitabayashi Y., Kudo T., Tsuboi H., Yamada T., Xu D., Shibata M., Matsumura D., Hayashi Y., Syamsul M., Inaba M. (2017). Normally-off C–H Diamond MOSFETs with partial C–O channel achieving 2-kV breakdown voltage. IEEE Electron. Device Lett..

[199] Fei W., Inaba M., Hoshino H., Tsuyusaki I., Kawai S., Iwataki M., Kawarada H. (2019). Point-arc remote plasma chemical vapor deposition for high-quality single-crystal Diamond selective growth. Phys. Status Solidi (A).

[200] Fei W., Bi T., Iwataki M., Imanishi S., Kawarada H. (2020). Oxidized Si terminated diamond and its MOSFET operation with SiO_2_ gate insulator. Appl. Phys. Lett..

[201] Geis M.W., Gregory J.A., Pate B.B. (1991). Capacitance-voltage measurements on metal-SiO_2_-diamond structures fabricated with (100)- and (111)-oriented substrates. IEEE Trans. Electron. Devices.

[202] Liu J., Ohsato H., Wang X., Liao M., Koide Y. (2016). Design and fabrication of high-performance diamond triple-gate field-effect transistors. Sci. Rep..

[203] Saha N.C., Kasu M. (2019). Heterointerface properties of diamond MOS structures studied using capacitance–voltage and conductance–frequency measurements. Diam. Relat. Mater..

[204] Schenk A.K., Sear M.J., Dontschuk N., Tsai A., Rietwyk K.J., Tadich A., Cowie B.C.C., Ley L., Stacey A., Pakes C.I. (2020). Development of a silicon–diamond interface on (111) diamond. Appl. Phys. Lett..

[205] Bi T., Niu J., Oi N., Inaba M., Sasaki T., Kawarada H. (2020). Application of 2DHG Diamond p-FET in cascode with normally-OFF operation and a breakdown voltage of over 1.7 kV. IEEE Trans. Electron. Devices.

[206] Zhu X.H., Bi T., Yuan X.L., Chang Y.H., Zhang R.M., Fu Y., Tu J.P., Huang Y.B., Liu J.L., Li C.M. (2022). C-Si interface on SiO_2_/(111) diamond p-MOSFETs with high mobility and excellent normally-off operation. Appl. Surf. Sci..

[207] Bi T., Chang Y., Fei W., Iwataki M., Morishita A., Fu Y., Niikura N., Kawarada H. (2021). C–Si bonded two-dimensional hole gas diamond MOSFET with normally-off operation and wide temperature range stability. Carbon.

[208] Fu Y., Kono S., Kawarada H., Hiraiwa A. (2022). Electrical Characterization of Metal/Al_2_O_3_/SiO_2_/Oxidized-Si-Terminated (C-Si-O) Diamond Capacitors. IEEE Trans. Electron. Devices.

[209] Kawarada H., Ota K., Fu Y., Narita A., Zhu X., Hiraiwa A., Fujishima T. Oxidized Silicon Terminated Diamond p-MOSFETs with Channel Mobility >150 cm^2^V^−1^s^−1^ and |VTH| > 3V Normally-off for Complementary Power Circuits. Proceedings of the 2023 International Electron Devices Meeting (IEDM).

[210] Sun S.C., Plummer J.D. (1980). Modeling of the on-resistance of LDMOS, VDMOS, and VMOS power transistors. IEEE Trans. Electron. Devices.

[211] Prins J.F. (1982). Bipolar transistor action in ion implanted diamond. Appl. Phys. Lett..

[212] Kato H., Makino T., Ogura M., Takeuchi D., Yamasaki S. (2013). Fabrication of bipolar junction transistor on (001)-oriented diamond by utilizing phosphorus-doped n-type diamond base. Diam. Relat. Mater..

[213] Cho S.J., Liu D., Hardy A., Kim J., Gong J., Herrera-Rodriguez C.J., Swinnich E., Konstantinou X., Oh G.-Y., Kim D.G. (2020). Fabrication of AlGaAs/GaAs/diamond heterojunctions for diamond-collector HBTs. AIP Adv..

[214] Hoshino Y., Kato H., Makino T., Ogura M., Iwasaki T., Hatano M., Yamasaki S. (2012). Electrical properties of lateral p–n junction diodes fabricated by selective growth of n+ diamond. Phys. Status Solidi (A).

[215] Suwa T., Iwasaki T., Sato K., Kato H., Makino T., Ogura M., Takeuchi D., Yamasaki S., Hatano M. (2016). Normally-off Diamond junction field-effect transistors with submicrometer channel. IEEE Electron. Device Lett..

[216] Shimada Y., Kato K., Ikeda S., Yoshida H. (1982). Low input capacitance and low loss VD-MOSFET rectifier element. IEEE Trans. Electron. Devices.

[217] Chen X.B., Huang M.M. (2012). A Vertical Power MOSFET with an Interdigitated Drift Region Using High-Insulator. IEEE Trans. Electron. Devices.

[218] Chang H.R., Holroyd F.W. (1990). High voltage power MOSFETs with a trench-gate structure. Solid State Electron..

[219] Inaba M., Muta T., Kobayashi M., Saito T., Shibata M., Matsumura D., Kudo T., Hiraiwa A., Kawarada H. (2016). Hydrogen-terminated diamond vertical-type metal oxide semiconductor field-effect transistors with a trench gate. Appl. Phys. Lett..

[220] Oi N., Inaba M., Okubo S., Tsuyuzaki I., Kageura T., Onoda S., Hiraiwa A., Kawarada H. (2018). Vertical-type two-dimensional hole gas diamond metal oxide semiconductor field-effect transistors. Sci. Rep..

[221] Iwataki M., Oi N., Horikawa K., Amano S., Nishimura J., Kageura T., Inaba M., Hiraiwa A., Kawarada H. (2020). Over 12000 A/cm^2^ and 3.2 m Ω cm^2^ Miniaturized Vertical-Type Two-Dimensional Hole Gas Diamond MOSFET. IEEE Electron. Device Lett..

[222] Aleksov A., Vescan A., Kunze M., Gluche P., Ebert W., Kohn E., Bergmeier A., Dollinger G. (1999). Diamond junction FETs based on δ-doped channels. Diam. Relat. Mater..

[223] Iwasaki T., Yaita J., Kato H., Makino T., Ogura M., Takeuchi D., Okushi H., Yamasaki S., Hatano M. (2014). 600 V Diamond Junction Field-Effect Transistors Operated at 200 °C. IEEE Electron. Device Lett..

[224] Iwasaki T., Kato H., Yaita J., Makino T., Ogura M., Takeuchi D., Okushi H., Yamasaki S., Hatano M. Current enhancement by conductivity modulation in diamond JFETs for next generation low-loss power devices. Proceedings of the 2015 IEEE 27th International Symposium on Power Semiconductor Devices & IC’s (ISPSD).

[225] Iwasaki T., Kato H., Makino T., Ogura M., Takeuchi D., Yamasaki S., Hatano M. (2017). High-temperature bipolar-mode operation of normally-off Diamond JFET. IEEE J. Electron. Devices Soc..

[226] Pham T.T., Pernot J., Masante C., Eon D., Gheeraert E., Chicot G., Udrea F., Rouger N. 200V, 4MV/cm Lateral Diamond MOSFET. Proceedings of the 2017 IEEE International Electron Devices Meeting (IEDM).

[227] Guo Z.B., Chow T.P. (2020). Performance projection of high-voltage, quasi-lateral diamond MOSFET for power electronics applications. Diam. Relat. Mater..

[228] Oi N., Kudo T., Inaba M., Okubo S., Onoda S., Hiraiwa A., Kawarada H. (2019). Normally-OFF Two-Dimensional Hole Gas Diamond MOSFETs Through Nitrogen-Ion Implantation. IEEE Electron. Device Lett..

[229] Liu B., Bi T., Fu Y., Kudara K., Imanishi S., Liu K., Dai B., Zhu J., Kawarada H. (2022). MOSFETs on (110) C–H Diamond: ALD Al_2_O_3_/Diamond interface analysis and high performance normally-OFF operation realization. IEEE Trans. Electron. Devices.

[230] Fu Y., Chang Y., Zhu X., Xu R., Xu Y., Kawarada H. (2022). Normally-off oxidized Si-terminated (111) Diamond MOSFETs via ALD-Al_2_O_3_ gate insulator with drain current density over 300 mA/mm. IEEE Trans. Electron. Devices.

[231] Tsunoda J., Niikura N., Ota K., Morishita A., Hiraiwa A., Kawarada H. (2022). 580 V Breakdown Voltage in Vertical Diamond Trench MOSFETs with a P Drift Layer. IEEE Electron. Device Lett..

[232] Kato H., Oyama K., Makino T., Ogura M., Takeuchi D., Yamasaki S. (2012). Diamond bipolar junction transistor device with phosphorus-doped diamond base layer. Diam. Relat. Mater..

[233] Perez G., Lefranc P., Jeannin P.-O., Eon D., Rouger N. Parallel and interleaved structures for diamond Schottky diodes. Proceedings of the 2017 19th European Conference on Power Electronics and Applications (EPE’17 ECCE Europe).

[234] Perez G., Letellier J., Maréchal A., Eon D., Chicot G., Jeannin P.O., Rouger N., Schanen J.L. Diamond Schottky barrier diodes for power electronics applications. Proceedings of the 2018 IEEE Energy Conversion Congress and Exposition (ECCE).

[235] Ren C., Malakoutian M., Li S., Ercan B., Chowdhury S. (2021). Demonstration of monolithic polycrystalline Diamond-GaN complementary FET technology for high-temperature applications. ACS Appl. Electron. Mater..

[236] Nagase M., Umezawa H., Shikata S. (2013). Vertical Diamond Schottky Barrier Diode Fabricated on Insulating Diamond Substrate Using Deep Etching Technique. IEEE Trans. Electron. Devices.

[237] Liu L., Wang J., Ren N., Guo Q., Sheng K. (2024). 1.43 kV 4H-SiC Lateral Junction Barrier Schottky Diode with High BFOM (390 MW/cm^2^). IEEE Electron. Device Lett..

[238] Wang Q., Hua H., Zheng L., Feng J.H., Zhang C., Gao M.Y., Qiu K., Luo J., Cheng X.H. (2024). Simultaneous improvement of high-frequency and Baliga figures of merit of 1.7 kV 4H-SiC MOSFET with retrograded JFET doping. J. Phys. D Appl. Phys..

[239] Liu X.K., Lin F., Li J., Lin Y.H., Wu J.Y., Wang H.F., Li X.H., Huang S.W., Wang Q., Chiu H.C. (2022). 1.7-kV Vertical GaN-on-GaN Schottky Barrier Diodes With Helium-Implanted Edge Termination. IEEE Trans. Electron. Devices.

[240] Wang C.L., Yan Q.L., Su C.X., Alghamdi S., Ghandourah E., Liu Z.H., Feng X., Zhang W.H., Dang K., Wang Y.M. (2023). Demonstration of the β-Ga_2_O_3_ MOS-JFETs with Suppressed Gate Leakage Current and Large Gate Swing. IEEE Electron. Device Lett..

[241] Zhang J., Dong P., Dang K., Zhang Y., Yan Q., Xiang H., Su J., Liu Z., Si M., Gao J. (2022). Ultra-wide bandgap semiconductor Ga_2_O_3_ power diodes. Nat. Commun..

[242] Wang H.C., Lumbantoruan F.J., Hsieh T.E., Wu C.H., Lin Y.C., Chang E.Y. (2018). High-Performance LPCVD-SiN/InAlGaN/GaN MIS-HEMTs with 850-V 0.98-mΩ·cm for Power Device Applications. IEEE J. Electron. Devices.

[243] Eon D. (2024). Diamonds in the Current: Navigating Challenges for the Integration of Diamond in Power Electronics. Phys. Status Solidi A.

[244] Benipal M.K., Brown J., Koeck F., Zaniewski A., Ahmad M.F., Nemanich R. Commercialization of Diamond Semiconductor Devices. Proceedings of the 2021 IEEE Energy Conversion Congress and Exposition (ECCE).

[245] Global Diamond Semiconductor Substrates Market Research Report—Segmentation by Type of Diamond Material (Synthetic Diamond, Natural Diamond); By Application (Diamond Detectors, Optical Systems, Power Electronics, Heat Spreaders, Others); By Manufacturing Process (Chemical Vapor Deposition (CVD), High-Pressure High Temperature (HPHT)); By Diamond Grade (Single Crystal Diamond, Polycrystalline Diamond); By End-User Industry (Consumer Electronics, Automotive, Industrial, Energy Sector, Telecommunications, Aerospace & Defense, Healthcare, Others); Region–Forecast (2024–2030). https://virtuemarketresearch.com/report/diamond-semiconductor-substrates-market.

[246] Diamfab Announces €8.7M Round of Funding from Asterion Ventures, Bpifrance and FondsRégional Avenir Industrie Auvergne-Rhône-Alpes. https://diamfab.com/wp-content/uploads/2024/03/20240328-PR-Diamfab-announced-a-E8.7M-round-of-funding.pdf.

[247] Diamond Quanta to Announce Findings on Novel Diamond Semiconductor Fabrication and Doping Techniques. https://thequantuminsider.com/2024/06/10/diamond-quanta-to-announce-findings-on-novel-diamond-semiconductor-fabrication-and-doping-techniques/.

